# Effectiveness of an action-oriented educational intervention in ensuring long term improvement of knowledge, attitudes and practices of community health workers in maternal and infant health: a randomized controlled study

**DOI:** 10.1186/s12909-018-1332-x

**Published:** 2018-09-27

**Authors:** Tereza Rebecca de Melo e Lima, Paula Ferdinanda Conceição de Mascena Diniz Maia, Emanuelle Pessa Valente, Francesca Vezzini, Giorgio Tamburlini

**Affiliations:** 1 Instituto de Medicina Integral Prof. Fernando Figueira (IMIP), Rua dos Coelhos, 300, Boa Vista, Recife, 50070-550 Pernambuco Brazil; 2 Faculdade Pernambucana de Saúde (FPS), Avenida Mal. Mascarenhas de Morais, 4861, Imbiribeira, Recife, 51150-000 Pernambuco Brazil; 3 Universidade Federal de Pernambuco, Av. Professor Moraes Rêgo, s/n. – Cidade Universitária, Recife, Pernambuco Brazil; 4 Università degli studi di Trieste - Piazzale Europa, 1, 34128 Trieste, Italy; 5 Centro Per la Salute del Bambino - Via De Rin, 19, 34143 Trieste, Italy

**Keywords:** Community health workers, Maternal and child health, Home visits, Primary health care, In-service training, Interactive training, Action-oriented training

## Abstract

**Background:**

The potential role of Community Health Workers (CHWs) in improving maternal and child health outcomes, particularly in low and middle-income countries and in disadvantaged communities, is receiving increased attention. Adequate and focused training is among the key requisites for enhancing CHWs performances and research is necessary to identify effective training methods.

**Methods:**

A randomized controlled study was designed to assess the effectiveness of a training course in improving knowledge, attitudes and practices (KAP) of CHWs regarding maternal and infant health. Seventy-eight CHWs belonging to Family Health Units in the city of Recife, Brazil were randomly allocated to intervention and control groups. The intervention group took part in a four-day interactive training course based on an action-oriented guide to perform home visits to pregnant women and their infants throughout pregnancy and infancy until 9 months of age. KAP in intervention group after training and after 1 year were compared to control group and to baseline.

**Results:**

Fifty-nine CHWs completed all KAP assessments (31 in intervention and 28 in control group). Baseline characteristics were similar in both groups. At 1 year from training, the intervention group had higher overall KAP score (120.65 vs. 108.19, *p* <  0.001) as well as knowledge (47.45 vs. 40.54, *p* <  0.001), practice (53.45 vs. 49.11, *p* <  0.001) and attitudes scores (19.74 vs. 18.81, *p* = 0.047) than the control group. Moreover, at 1 year from training, the intervention group maintained significant improvements in overall KAP score (120.65 vs. 106.55, *p* <  0.001) as well as in knowledge (45.45 vs. 42.13, *p* <  0.001), and practice (53.45 vs. 45.29, *p* <  0.001) scores with respect to baseline. In the control group, overall KAP (106.59 vs. 108.19, *p* = 0.345) as well as separate knowledge, attitudes and practices scores remained unchanged.

**Conclusions:**

A four-day interactive training course on action-oriented home visits to pregnant women and infants produced a sustained improvement of CHWs’ KAP and may represent a model to ensure retention of acquired competences.

**Trial registration:**

RBR-9gchqr. Date registered: July 21, 2018 (Retrospectively registered).

**Electronic supplementary material:**

The online version of this article (10.1186/s12909-018-1332-x) contains supplementary material, which is available to authorized users.

## Background

The potential role of Community Health Workers (CHWs) in improving maternal and child health outcomes, particularly in low and middle-income countries and in disadvantaged communities, has been receiving increased attention [1–10].

Recognizing this potential implies guaranteeing adequate selection and appropriate training, support and supervision to CHWs [11]. However, the training and responsibilities of CHWs vary widely in different countries and there is still the need for research to identify effective approaches for CHWs recruitment, training and for an effective tasks shifting, as well as efforts to improve their competencies, motivation and productivity [5, 12–15].

CHWs are usually defined as community members chosen by their community to support or provide health interventions at household level, which are usually linked to the health system, but have shorter training than professional health workers [16]. In Brazil, CHWs (Agentes Comunitários de Saúde – ACSs, in Portuguese) are crucial actors of the Family Health Strategy, a key component of the universal health care system of Brazil (Brazilian Unified Public Health System - SUS) [17].

CHWs were officially established as health professionals by Ministry of Health of Brazil in 1991, building on the experience of the Program of Community Health Workers (PACS) in the late 80s [18]. CHWs are community members of Family Health Teams (FHT), who live in the area in which they work. They are hired through public selections that require at least primary education and a mandatory basic training of a minimum of 40 h, and are paid by the municipal health authority. The Ministry of Health recommends that each CHW should be in charge of an average of 750 individuals (150 households), including men, women and children in all phases of life [17].

The most important task of the CHW is the home visit (HV) [19]. According to the National Policy of Primary Care of Brazil (PNAB), it is responsibility of CHWs, among others, to follow, through home visits, all families and individuals [17]. The PNAB recommends that the visits should be planned keeping, as a reference’ standard, the average of one visit/family/month but considering risk and vulnerability criteria, so that more visits are made to families with the greatest needs.

In this context, CHWs must perform monthly HVs to all families of the area, to provide health information, prevent disease and promote health, identify problems and refer it to health services, ultimately acting as a bridge between the health services and the community. In order to do this effectively, HVs must be based on clear goals, adequate training and guidance [20, 21].

However, current recommendations fail to provide satisfactory guidance for HVs. Recommendations are generic and limited to setting a minimum amount of visits that should be carried out. Moreover, studies have observed: the lack of proper definition of CHWs’ tasks [22]; a greater focus on illness (consultations, delivery of drugs and lab test results) than on health promotion [21, 23]; little integration among FHT staff [24]; insufficient communication with families [25]; and excess of bureaucratic tasks [22, 26]. Inadequate training is recognized as a major obstacle to effective HV performance [24, 27, 28].

Although HVs performed by CHWs have been identified as key to achieve improved maternal, neonatal and child health (MNCH) [29, 30] and child development [4, 31], official national guidelines and training materials for CHWs do not indicates specific tasks and referral criteria for specific prenatal periods and developmental stages during early infancy [32].

Building on the evidence on the existing performance and training gaps, we developed and conducted an action-oriented educational intervention and assessed its effectiveness in ensuring sustained improvement of knowledge, attitudes and practices of CHWs regarding prenatal and postnatal HVs.

## Methods

### Study design and aims

This was an educational intervention study, randomized and controlled, to evaluate the effectiveness of an action-oriented training on knowledge, attitudes and practices (KAP) of community health workers (CHWs) regarding maternal and infant health.

The study design was based on the classic Kirkpatrick’s four levels model for evaluation of training programs [33]. In this paper, we focused on level 2 (learning), and measured to which extent knowledge, attitudes and practice were improved and retained. An educational intervention was applied and pre-, post-, and one-year follow-up KAP assessments were performed from October 2015 to December 2016.

### Study setting and participants

The study was carried out in the city of Recife, which has 1.633.697 habitants and is the capital of Pernambuco, a State in northeastern Brazil. In 2015, the infant mortality rate in Recife was 7.5 deaths of children under 1 year per 1000 live births, the neonatal mortality rate was 16 newborn deaths per 1000 live births, and maternal mortality ratio was 80.3 maternal deaths per 100.000 live births.

There are 125 Family Health Units (FHUs) in Recife, distributed in 6 different political-administrative regions called Health Districts (HDs). Each FHU includes one or two Family Health Teams (FHTs) composed by at least: a physician, a nurse, a nursing technician and four to six CHWs.

The study population consisted of CHWs belonging to a group of 12 FHUs of 3 different HDs that are co-managed by the municipal health authority of Recife and the Institute of Integral Medicine Professor Fernando Figueira (IMIP), a non-profit organization that operates in the areas of medical and social assistance, teaching, research and outreach programs and is accredited by the Ministry of Health as a National Referral Center for Mother and Child Care Programs.

The 12 FHUs include 18 FHTs, with a total of 86 CHWs. These teams assist a population of about 70.000 inhabitants (11.500 families) of 10 low-income communities. In their catchment area, 7 maternal deaths and 96 infant deaths were reported in 2015, representing 36% of maternal deaths and 54% of infant deaths from the whole city of Recife.

Inclusion criteria for participation of this study were CHWs working in FHTs that are co-managed by IMIP. Exclusion criteria were CHWs who were on vacation or on any type of leave during the data collection periods.

### Procedures, recruitment and randomization

All CHWs meeting the inclusion criteria were invited to participate in the study throughout personal invitation and were informed about its purpose and their voluntary participation. The participants who accepted to participate signed a written informed consent prior to any involvement in the study.

The 18 FHTs underwent paired randomization, and were divided into two groups (intervention and control) matched by main personal and catchment area characteristics. Each FHT was assumed as a block (with 4 to 6 CHWs each) and was paired with a neighbor FHT with similar population characteristics. To minimize contamination among CHWs belonging to the same FHT, each block of two FHTs was randomly allocated to either intervention or control group. Thus, each group included 9 FHTs.

CHWs of the intervention group were invited to participate in the training. As the intervention was an educational training, it was not possible to blind the participants. The control group also received the training, but after the study was finalized.

### Educational intervention

The educational intervention consisted of a training course for action-oriented home visits to pregnant women and their infants. The course objectives and materials, including an action-oriented guide, were designed collaboratively by a multidisciplinary group of experienced professionals in health education and maternal, neonatal and child health, based on updated international and national recommendations on prenatal and postnatal HVs carried out by CHWs, and on emerging training needs, according to classic instructional design methods [34, 35].

The *Training Course on Home Visits to Pregnant Women and Infants* was informed by pedagogy of autonomy and problem-based active methodologies [36–39]. Case studies focused on participants experiences were used with active methods of learning followed by simulations, interactive lectures and small group activities, ultimately requiring 32 h (4-days). These approaches were chosen because they can stimulate the critical-reflexive knowledge and the capacity to detect real problems and search for solutions as well as to strengthen horizontal competences such as communication and teamwork [40–43]. To ensure the integration of FHT and provide support to the new tasks proposed for CHWs, a 16-h course covering the same contents was offered to physicians and nurses belonging to the same FHTs.

The main contents addressed by the training included: assessment of mother’s and infant’s health and wellbeing, information on preventive practices and prenatal visits, information on care for labor and delivery, anticipatory advice on maternal and neonatal most common postpartum problems, essential care of the newborn, support to breastfeeding, nutrition, accident prevention and immunization, evaluation of infant development, promotion of practices that favor parent-child interaction, assessment of family environment, identification of socioeconomic and psychosocial problems and effective communication with parents and family.

The course was based on the *Guide for Innovative Home Visits to Pregnant Women and Infants* [44], which provides action-oriented instructions for each home visit, for a total of 10 visits, including 5 visits for the mother from conception to childbirth and postpartum period and 5 visits focusing on maternal health and on child health and development until the age of 9 months. Tasks related to each visit were detailed and included what should be asked, observed and identified by CHWs, and actions to be taken according to a three-level risk classification. This structure followed the model of the Integrated Management of Childhood Illness (IMCI) [45].

The course took place in November 2015. Between November 2015 and November 2016, trainers were available for support throughout personal visits and by phone in case of doubts and difficulties about the use of the guide for HVs.

Efforts were made to minimize the training costs to CHWs and to system (including venue, teachers’ salary, printed materials, food and transportation), in order to facilitate transferability of the intervention in other low and middle-income countries (LMIC), improving its external validity. Meetings with managers (Coordination of Districts and Municipal Health Department) were also held in order to ensure their support to the intervention.

### Data collection

The data were collected at three different times: before training (t0), immediately after training (t1), and 1 year after training (t2). Knowledge (K), Attitudes (A) and Practices (P) of intervention and control groups were assessed at t0 and t2. Knowledge of intervention group was also collected at t1 to assess instant change of knowledge.

The KAP of the participants were assessed through a written questionnaire, which was developed taking into account both the competences that the training was supposed to strengthen as well as the newly introduced ones.

The questionnaire addressed 8 domains: 1) recognition of risk factors in pregnant women, 2) recognition of warning signs in pregnant women, 3) recognition of risk factors and warning signs in young infants 4) antenatal care routine, 5) newborn care routine, 6) child care routine, 7) child development, 8) aims of home visits to mother and child health.

To optimize semantic, face and content validity, the draft questionnaire was distributed to researchers in health education, primary care professionals (including CHWs), pediatricians and gynecologists-obstetricians for a critical appraisal of technical content, relevance to CHWs’ job and clarity of wording.

To ensure validity of construct and reproducibility, a pilot study was conducted with 20 CHWs who were not participating in this study. The same subjects filled in the questionnaires in two separate moments with an interval of 1 week in order to assess reliability. The responses were analyzed throughout measurement of agreement between responses (test-retest). The comparison of test and retest values (intraclass correlation) with Student t showed a high index of concordance between responses in 99.7% of the questionnaire’s items. The Kappa coefficient (interrater correlation) reached a moderate to almost perfect level of agreement in 88% of the analyzed questions, being 13% with moderate agreement (0.40–0.59), 38% with substantial agreement (0.60–0.79) and 38% with almost perfect agreement (0.80–1.00).

After revision, the final version of the questionnaire was developed (Additional file 1). It was divided in four sections: Section I included socio-demographic and professional experience, previous participation in training courses, number of pregnant women and children visited per month and duration of these home visits. Section II covered Knowledge assessment with 20 questions, being 2 open-ended questions and 18 multiple-choice questions. Out of the 18 multiple-choice questions, 11 included 4 alternatives with only one correct answer each, addressing the maternal and child health content recommended by the Ministry of Health, each correct answer generating a score of 1, for a maximum of 11 points. The other 7 questions of this section considered a total of 50 alternative answers (4 questions with 5 alternatives each and 3 questions with 10 alternatives each). These 50 alternatives were scored for a maximum of 50 points. Thus, the maximum total score for this section was 61 points. Section III focused on the Attitudes self-Assessment, consisting of a list of 20 statements describing attitudinal behaviors (such as punctuality and assiduity, communication, relationship, reflection on work process, interest in and satisfaction with work), to be answered on a 5-point Likert scale, where 1 is *totally disagree* and 5 is I *totally agree* with the described behavior. Answers 4 (*agree*) and 5 (*totally agree*) were considered as correct answers and rated 1 point each. Thus, each statement could generate a maximum score of 1. Accordingly, the maximum total score for this section was 20 points. Section IV consisted of Practices self-Assessment including a list of 60 recommended tasks to be performed by CHWs during HVs (25 tasks in pregnancy care and 35 tasks in postnatal mother and child care). Answers included a 5-point frequency scale where 1 is *never* and 5 is *always* according to how often each task were performed. Answers corresponding to 4 (*often*) and 5 (*always*) were considered as correct and rated 1 point. Hence, each task could generate a maximum score of 1. Therefore, the highest total score for this section was 60 points. Overall, the maximum total score for the three sections was 141 points (KAP score). A percentage of at least 70% of correct answers was considered as satisfactory. This choice was based on the academic grading system commonly used by the education system in Brazil, which adopts passing criteria ranging from 60 to 70%.

### Outcome measures

The primary outcome was the increase and retention in the average overall KAP score at one-year follow up as compared to baseline and to control group. Secondary outcomes were the increase in the average score and in percentage of correct answers in each section (K, A and P), and in the percentage of CHWs who improved their score, overall and in K, A and P section after training.

### Evaluation design and data analysis

We compared overall KAP scores and separate K, A and P scores and percentage of correct answers of the intervention group at 1 year after training (t2) to scores of the same group at baseline (t0) and of the control group at baseline and after 1 year. We also compared K scores and percentage of correct answers of the intervention group immediately after training (t1) to scores of the same group at baseline.

The Statistical Package for the Social Sciences (SPSS) 13.0 for Windows and Microsoft Excel 2010 were used for statistical analysis. All tests were applied with 95% of confidence. The results are presented in table form with their respective absolute and relative frequencies. Numerical variables are represented by measures of central tendency and dispersion measures, described as mean (± standard deviation). Kolmogorov-Smirnov Normality Test was used for quantitative variables. Student’s t-test (normal distribution) and Mann-Whitney (non-normal distribution) were used to the comparisons of two groups. Paired student’s t-test (normal distribution) and Wilcoxon (non-normal distribution) were used to paired groups.

## Results

**Fig. 1:**
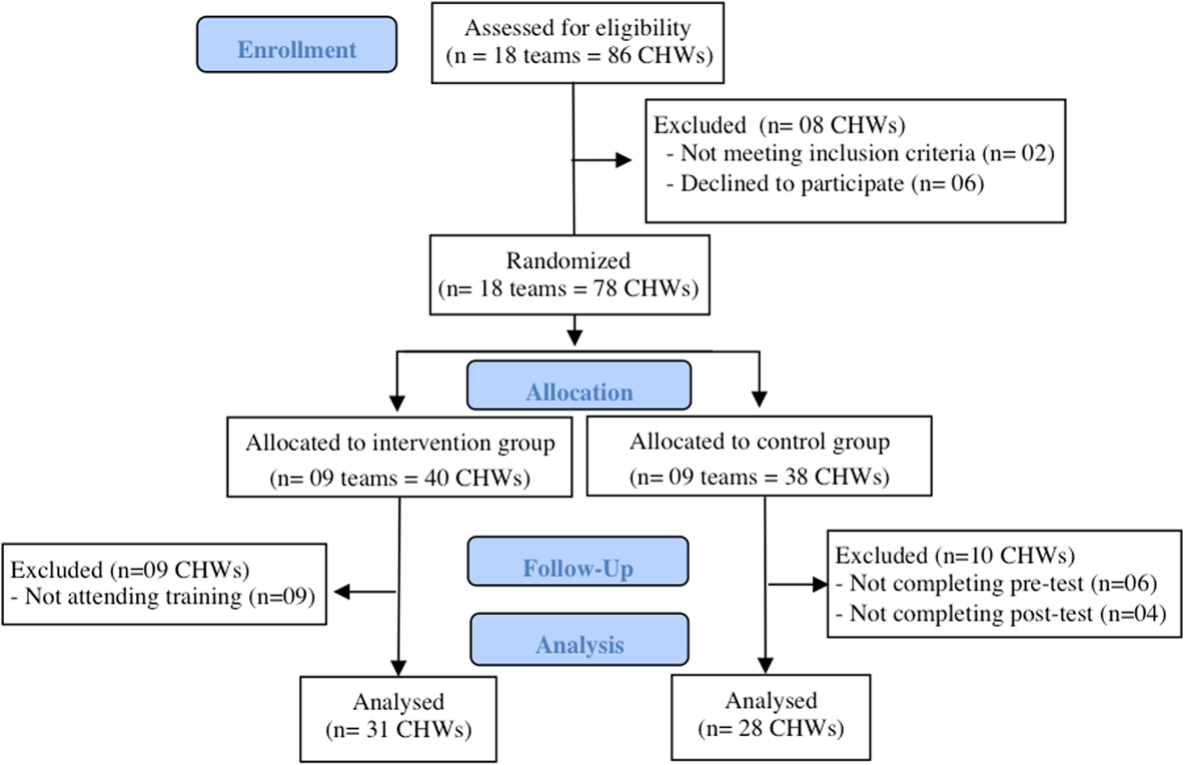
Flow diagram of participants. The process of enrollment, allocation, follow-up and analysis of outcomes

Of the 86 CHWs eligible, 78 (90.7%), 40 in the intervention group and 38 in the control group, ultimately took part in the study. Reasons for not participating were: annual leave (1) and medical reasons (1), while 6 declined to participate. At follow-up, 31 CHWs of the intervention group completed the training and all assessments, and 28 CHWs of control group completed all assessments, for a total of 59 participants who completed the study (Fig. 1).

### Baseline socio-demographic and work characteristics

The mean age of the 59 CHWs was 44.96 years (± 10.05 years), the majority were women (96.5%) and over half had completed high school (74.5%). The mean length of working experience as CHWs was 16.67 years (± 8.06 years). A percentage of 52.5% and 72.9% of participants reported previous training in pregnancy health and child health, respectively. Only 11.9% reported previous training in home visits to pregnant women and infants. Fifty-seven CHWs (96.6%) declared interest in attending trainings.

Each CHW assisted a mean of 194.15 (± 32.81) families and visited monthly at home a mean of 5.70 (± 2.86) pregnant women and 8.55 (± 4.67) children under 1 year. A mean of 8.19 (± 2.67) home visits was made to each pregnant woman during pregnancy, with duration of 26.79 (± 8.23) minutes. A mean of 8.48 (± 3.52) home visits were made to each child under 9 months, with 26.25 (± 8.83) minutes of duration each. There were no significant differences in the baseline characteristics of intervention and control group (Table 1).

**Table 1:** Baseline characteristics of Community Health Workers (*n* = 59)

		Groups		
Characteristics	Total (*n* = 59)	Intervention (*n* = 31)	Control (*n* = 28)	*p*-value
	n(%)	n (%)	n (%)	
Gender				1.000^a^
Male	2 (3.5)	1 (3.4)	1 (3.6)	
Female	55 (96.5)	28 (96.6)	27 (96.4)	
Educational level				1.000^a^
Elementary school	3 (5.5)	2 (7.1)	1 (3.7)	
High school	41 (74.5)	20 (71.4)	21 (77.8)	
Higher education	11 (20.0)	6 (21.4)	5 (18.5)	
Previous training in pregnant health	31 (52.5)	16 (51.6)	15 (53.6)	0.880^b^
Previous training in child health	43 (72.9)	22 (71.0)	21 (75.0)	0.728^b^
Previous training in home visits	7 (11.9)	3 (9.7)	4 (14.3)	0.698^a^
Have interest in attending training	57 (96.6)	30 (96.8)	27 (96.4)	1.000^a^
Have difficult to attend trainings	16 (27.1)	10 (32.3)	6 (21.4)	0.350^b^
	Mean ± *SD*	Mean ± *SD*	Mean ± *SD*	
Age (years)	44.96 ± 10.05	45.96 ± 11.22	43.93 ± 8.76	p-value
Years of experience as CHW	16.67 ± 8.06	18.58 ± 9.14	14.96 ± 6.68	0.457^c^
Years of work in the same FHU	14.54 ± 7.86	15.38 ± 9.46	13.63 ± 5.73	0.110^c^
Number of families assisted	194.15 ± 32.81	189.84 ± 28.82	198.93 ± 36.67	0.411^c^
Number of pregnant visited per month	5.70 ± 2.867	4.89 ± 2.18	7.09 ± 3.44	0.292^c^
Number of home visits in pregnancy	8.19 ± 2.67	7.96 ± 2.78	9.53 ± 2.54	0.077^c^
Duration of home visit to pregnant (minutes)	26.79 ± 8.23	25.35 ± 8.91	28.57 ± 7.09	0.359^d^
Number of children under 1 year visited a month	8.55 ± 4.67	7.90 ± 4.45	9.54 ± 4.99	0.271^d^
Number of home visits to children until 9 months	8.48 ± 3.52	9.07 ± 4.63	7.84 ± 1.51	0.333^c^
Duration of home visit to child (minutes)	26.25 ± 8.83	24.20 ± 8.97	28.95 ± 8.09	0.078^d^

(a) Fisher’s Exact Test (b) Chi-Square Test (c) Student’s t-test (d) Mann-Whitney test

### Pre-training assessments of knowledge, attitudes and practices

#### Overall KAP score

At the pre-training assessment, the average overall KAP score of intervention and control group were 106.55 (± 12.17) and 106.59 (± 8.57) respectively (*p* = 0.987), corresponding to a mean percentage of 79.9% and 77.9% of correct answers. Forty-two (71.2%) of the 59 participants pre-assessed attained more than 70% of correct answers.

#### Knowledge scores

At pre-training knowledge assessment, the average scores of intervention and control group were 42.13 (± 6.02) and 40.50 (± 4.33) respectively (*p* = 0.235), corresponding to a mean percentage of 63.9% and 60.9% of correct answers in this section. Twenty (33.8%) of the 59 participants pre-assessed attained more than 70% of correct answers in this section.

The domains with the lowest proportion of correct answers of all participants were: *recognition of risk factors for pregnant and child health* (mainly socioeconomic and psychosocial risk factors such as economic dependence [3.4%], marital problems [11.9%], low educational level [13.6%], low parental education [13.6%], parents’ financial problems [5.1%], death of a under 5 year sibling [13.6%]), *antenatal care* (vaccination in pregnancy [42.8%]), *child care* (prevention of accidents – sleeping position [55.9%]) and *child development* (recognition of risk factors for child development - parents’ low level of education [24.1%]).

Considering all 61 single items, there was no statistically significant difference in the number of correct answers between the intervention and the control groups with the exception of two items: *recognition of domestic violence (as a risk factor for child development)* (*p* = 0.048) and *reduction of preterm and low birth weight (as an aim of home visits)* (*p* = 0.034), which were both lower in control group. Number and percentage of correct answers of all single items in intervention and control groups are detailed in (Additional file 2: Table S1).

#### Attitude scores

In the pre-training attitude assessment, the average scores of intervention and control group were 19.13 (± 1.78) and 18.22 (± 2.03) respectively (*p* = 0.037), corresponding to 97.2% and 91.5% respectively of behaviors with which the participants agreed/totally agreed. In this section, all participants answered that agreed/totally agreed with at least 70% of the described behaviours.

Considering each behaviour separately, a significant difference in the number of correct answers between the intervention and the control groups was observed in only 1 among 20 behaviors: *know the health indicators of the population that assist* (*p* = 0.023), which was lower in control group. Number and percentage of behaviours with which participants agreed/totally agreed are detailed in (Additional file 2: Table S2).

#### Practice scores

In the pre-training assessment of practice, the average scores of intervention and control group were 45.29 (± 9.64) and 47.93 (± 6.53) respectively (*p* = 0.220), corresponding to 78.5% and 81.2% respectively, of tasks that were declared as performed often/always. Forty-four (74.6%) of the 59 participants attained more than 70% of correct answers in this section.

The tasks that were less frequently declared as performed often/always in home visits to pregnant women were: *encourage the father’s participation in prenatal care* (52.5%), *encourage the reading of the pregnant woman’s card* (42.3%), *inform about iron replacement* (64.4%)*, orient about physical activity in pregnancy* (35.6%), *stimulate mother to sing for the baby in the belly* (33.9%), *stimulate the mother to talk to the baby in the belly* (62.7%), *explain about labor and childbirth* (52.5%), and *provide explanations about postpartum* (66.1%).

In postnatal home visits to mother and child, tasks that were less frequently declared as performed often/always were: *orient about weaning and storage of breast milk* (67.8%), *observe/evaluate the development of the child* (66.1%), *explain mother/caregiver about developmental stages (*66.1%), *stimulate the mother/caregiver to read to the child* (57.6%), *explain about the importance of reading for the child* (49.1%), *encourage to tell stories to the child* (61.0%), *encourage listening to music/sing with child* (45.7%), *stimulate using moments of routine as stimuli moment* (38.9%).

There was no statistically significant difference in the frequency of performance of the tasks between the intervention and the control groups, except in 2 out of 60 tasks: *stimulate the mother/caregiver to read to the child* (*p* = 0.029) and *encourage mother/caregiver to tell stories to the child* (*p* = 0.012*),* which were both lower in intervention group. Number and percentage of tasks that were declared as performed often/always are detailed in (Additional file 2: Table S3).

### Post-training assessments and effectiveness of intervention on knowledge, attitudes and practices

#### Overall KAP score

The average overall KAP score of intervention group increased from 106.55 (± 12.17) to 120.65 (± 9.55) (*p* < 0.001), between pre-training (t0) and one-year after training (t2) assessments, and the percentage of correct answers went from 79.9 to 87.2%. Twenty-eight (90.3%) participants of the intervention group improved their individual KAP score. The number of participants that attained more than 70% of correct answers increased from 21 (67.7%) to 29 (93.5%), between t0 and t2.

In the control group, the average overall KAP score remained unchanged from 106.59 (± 8.57) to 108.19 (± 10.55) (*p* = 0.345), between t0 and t2 assessments, corresponding to the mean percentage of 77.9% and 78.9% of correct answers. Moreover, at t2 assessment, a significant difference was observed between KAP score of intervention (120.65 ± 9.55) and control group (108.19 ± 10.55) (*p* < 0.001).

#### Knowledge scores

**Fig. 2:**
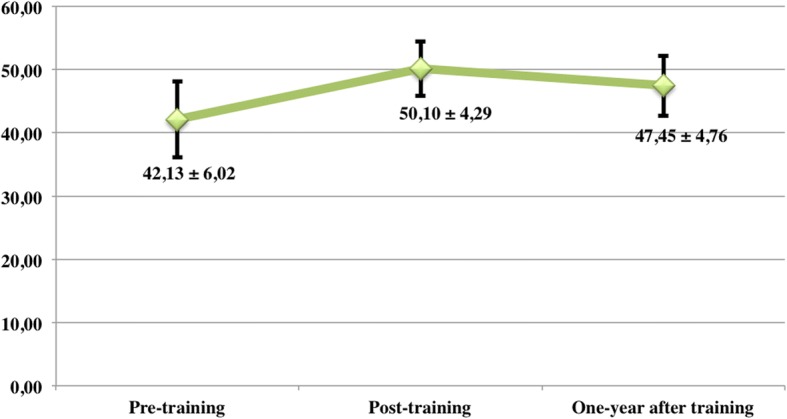
Average knowledge scores of intervention group (*n* = 31), comparing three different moments of assessment

The average knowledge score of intervention group improved immediately after the training (t1), increasing from 42.13 (± 6.02) at t0 to 50,10 (± 4.29) at t1 (*p* < 0.001), and the percentage of correct answers went from 63.9 to 78.9%. The knowledge score remained higher at t2 than at t0 (47.45 ± 4.76, *p* < 0.001), and correct answers decreased only slightly to 73.3%. The variations of average knowledge scores of intervention group at different time points are shown in Fig. 2.

All the 31 participants (100.0%) of the intervention group improved their individual knowledge score after training, and the number of participants that attained more than 70% of correct answers increased from 14 (45.1%) to 30 (96.7%) between t0 and t1, and decreased to 25 (77.5%) at t2, a figure still significantly higher (*p* < 0.001) than at t0. Moreover, at t2, a significant difference was observed between knowledge scores of intervention (47.45 ± 4.76 points) and control group (40.54 ± 4.07 points) (*p* < 0.001).

The increment in knowledge of intervention group at follow up remained statistically significant in the following items: *recognition of warning signs in pregnant women* (absence of fetal movements), *recognition of risk factors/warning signs in children* (parents’ financial problems, delay in development and early weaning); *antenatal care* (number of consultations and vaccination in pregnancy); *newborn care* (moment of first home visit and frequency of breastfeeding); *child care* (nutrition in the first 2 years and back to sleep position); *child development* (important stages for brain development, practices that stimulate child development such as musical experience and reading, recognition of risk factors for child development such as parental mental health problem, excessive use of digital devices) and *aims of home visits* (reduction of preterm and low birth weight and greater cognitive and emotional development). The number and percentage of correct answers of intervention group in each item assessed, comparing t0, t1 and t2, are described in (Additional file 2: Table S4).

In the control group, the average knowledge score remained unchanged from 40.50 (± 4.33) at t0 to 40.54 (± 4.07) (*p* = 0.764) at t2. The comparisons of number of correct answers between the two groups considering each item assessed in t0 and t2 are described in (Additional file 2: Table S5).

#### Attitude scores

The average attitude score of intervention group did not change, moving from 19.13 (± 1.78) at t0 to 19.74 (± 0.68) (*p* = 0.088) at t2 and the percentage of correct answers remained quite high (97.2% and 99.1% respectively). Similarly, the average score of control group remained unchanged, moving from 18.22 (± 2.03) to 18.81 (± 2.35) (p = 0.088) as well as the mean percentage of correct answers (91.5% and 94.1% respectively). At t2, however, a significant difference emerged between intervention (19.74 ± 0.68) and control group (18.81 ± 2.35) (*p* = 0.047).

The number and percentage of behaviours with which participants of intervention group agree/totally agree, at t0 and t2, are described in (Additional file 2: Table S6). The comparisons of number of behaviors with which participants agree/totally agree between the two groups are described in (Additional file 2: Table S7).

#### Practice scores

The average practices score of intervention group increased from 45.29 (± 9.64) to 53.45 (± 7.35) (*p* < 0.001), between t0 and t2, and the percentage of correct answers moved from 78.5 to 89.2%. Twenty-four out of 31 (77.4%) participants of intervention group improved their individual knowledge score after training, and the number of participants that attained more than 70% of correct answers increased from 20 (64.5%) to 28 (90.3%). At t2, a significant difference was observed between practice scores of intervention (53.45 ± 7.35) and control group (49.11 ± 8.20) (*p* = 0.036).

The improvement in reported practices in the intervention group was statistically significant in 7 out of 25 tasks related to care to pregnant women (*encourage the father’s participation in prenatal care, encourage the reading of the pregnant woman’s card, alert on alcohol and drug risks in pregnancy, explain about child development in pregnancy, stimulate mother to sing for the baby in the belly, stimulate the mother to talk to the baby in the belly,* and *orient about future contraception and family planning)* and in 7 out of 35 tasks related to postnatal care (*identify children at risk, identify signs of child suffering violence, identify problems in breastfeeding, orient about child meal times, identify problems in growth and development, encourage listening to music/sing with child,* and *stimulate using moments of routine as stimuli moment).* The number and percentage of tasks at t0 and t2 that were reported as performed often/always by intervention group, are described in (Additional file 2: Table S8).

In the control group, the average practice score remained unchanged between t0 and t2, moving from 47.93 (± 6.53) to 49.11 (± 8.20) (*p* = 0.540), corresponding to 81.2% and 82.1% of correct answers, respectively. The tasks that were declared as performed often/always in intervention and control group are described in (Additional file 2: Table S8).

**Fig. 3:**
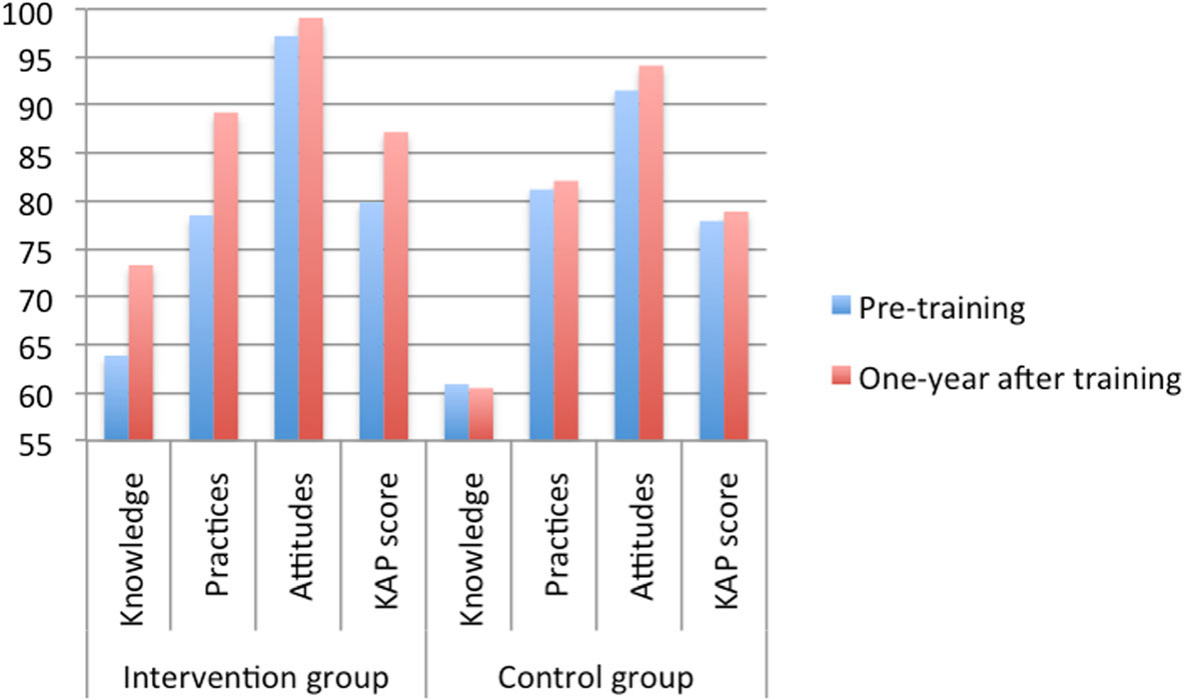
Percentage of correct answers of intervention and control group comparing pre- and one-year after training

Table 2 shows overall and separate K, A and P scores of intervention and control groups at t0 and t2. The comparisons between intervention and control group are described in Table 3. The change in the percentage of correct answers of intervention and control groups can be seen in Fig. 3.

**Table 2:** Overall and separate KAP scores of community health workers at t0 and t2, comparing moments

	Time		
Groups	t0 (Pre-training)	t2 (One-year after training)	*p*-value
	Mean ± SD	Mean ± SD	
Intervention group			
-Knowledge (0–61 points)	42.13 ± 6.02	47.45 ± 4.76	< 0.001*
-Practices (0–60 points)	45.29 ± 9.64	53.45 ± 7.35	< 0.001*
-Attitudes (0–20 points)	19.13 ± 1.78	19.74 ± 0.68	0.088**
-KAP score (0–141 points)	106.55 ± 12.17	120.65 ± 9.55	< 0.001*
Control group			
-Knowledge (0–61 points)	40.50 ± 4.33	40.54 ± 4.07	0.764**
-Practices (0–60 points)	47.93 ± 6.53	49.11 ± 8.20	0.540**
-Attitudes (0–20 points)	18.22 ± 2.03	18.81 ± 2.35	0.088**
-KAP score (0–141 points)	106.59 ± 8.57	108.19 ± 10.55	0.345**

(*)Paired student’s t-test (**) Wilcoxon

**Table 3:** Overall and separate KAP scores of community health workers in intervention and control groups

	Groups		
Groups	Intervention group	Control group	*p*-value
	Mean ± SD	Mean ± SD	
t0 (Pre-training)			
-Knowledge (0–61 points)	42.13 ± 6.02	40.50 ± 4.33	0.235*
- Practices (0–60 points)	45.29 ± 9.64	47.93 ± 6.53	0.220*
- Attitudes (0–20 points)	19.13 ± 1.78	18.22 ± 2.03	0.037**
- KAP score (0–141 points)	106.55 ± 12.17	106.59 ± 8.57	0.987*
t2 (One-year after training)			
- Knowledge (0–61 points)	47.45 ± 4.76	40.54 ± 4.07	< 0.001*
- Practices (0–60 points)	53.45 ± 7.35	49.11 ± 8.20	0.036*
- Attitudes (0–20 points)	19.74 ± 0.68	18.81 ± 2.35	0.047**
- KAP score (0–141 points)	120.65 ± 9.55	108.19 ± 10.55	< 0.001*

(*)Student’s t-test (**)Mann-Whitney

## Discussion

Our study was aimed to assess the effectiveness of an action-oriented training course in ensuring sustained improvement in knowledge, attitudes and practices of CHWs involved in home visits to pregnant women and their infants. Results indicate that CHWs belonging to the intervention group showed significant improvements in overall KAP scores, as well as in K, A and P scores when analyzed separately, with respect to control group. The intervention group showed significant improvements with respect to baseline in overall KAP score as well as in K and P scores. In the control group, overall KAP as well as separate K, A and P scores remained unchanged between baseline and follow up assessments. Improvements were also observed in the percentage of correct answers in each section, and in the percentage of CHWs who improved their score, overall and in each section.

Attitude and practice scores were higher than knowledge scores, both before and after training. This is not surprising as they were self-assessed and might be influenced by social desirability, which is quite common in experimental and survey findings in social sciences [46]. However, the improvement in scores observed in the intervention group was not observed in the control group, which could reflect a real practical improvement as a consequence of the intervention.

We also observed in the two groups a low proportion of correct answers for items regarding the recognition of risk factors for maternal and child health and child development, in spite of the fact that socioeconomic and psychosocial factors should be among the priority target of CHWs activities [47]. Some studies showed that CHWs who make home visits can learn to recognize danger signs during pregnancy and childhood illness and they can teach these to mothers, other caregivers, and family members [48–50]. However, few studies highlighted the importance of early recognition of social risk factors [22, 51] and, to our knowledge, no other studies assessed the effects of training CHWs in identification of socioeconomic and psychosocial risk factors and problems for pregnant and child health and child development during home visits.

Another important domain where results in knowledge and practices at pre-training assessment were poor was early child development, in spite of growing evidence on importance for establishing the biopsychosocial basis of health and development up to adult life [52], and the fact that globally CHWs are seen as key vehicles for early child development promotion [53].

Assessment and promotion of early child development was one of the main content innovations of the training course designed in this study, and the increase in knowledge and practices related to child development in the intervention group was significant after the training and remained such at follow-up.

We acknowledge that the number of participants in our study was limited. Still, it was sufficient to show significant differences, which we believe can be entirely attributed to the intervention because the baseline socio-demographic characteristics and the working experiences were comparable between intervention and control groups. Moreover, the participants presented the same socio-demographic profile observed in other studies with CHWs conducted in different regions of Brazil [22, 23, 26, 54–57].

We believe that an important determinant of the results was the training method, which was based on pedagogy of autonomy and problem-based active methodologies [36–39], taking into account well established methods of curriculum analysis and design [34, 58–60]. Other studies used similar approaches in training CHWs with positive results [43, 61, 62]. We believe that that the fact that CHWs received, as an essential component of the training, a guide that provides detailed action-oriented instructions for a total of 10 prenatal and postnatal visits, was key to establish a sound basis for more effective and efficient use of their time.

To our knowledge no other studies assessed the long-term retention of CHWs KAP regarding the content of home visits focusing on maternal and child health and child development in home visits. Although developed for the Brazilian context, our training approach, which is in line with WHO recommendations for the optimization of the roles and responsibilities of health workers through task shifting [63], could be adopted in other countries where CHWs play a role in primary care for mothers and infants.

We acknowledge that, even if the training proved to be effective in ensuring sustained improvement in knowledge and practice of CHWs, it may not be sufficient to improve the actual performance of CHWs, because there are many other factors affecting it. The quality of CHWs based interventions depends not only on appropriate training but also on supportive supervision and effective coordination within primary care services [1]. Moreover, complex system issues, including the self-attributed role of CHWs and their status vis-à-vis the other health professionals and the community may be difficult obstacles to overcome. And demand-side issues and other socially determined factors may hamper the impact of improved CHWs performance on family practices.

## Conclusions

A randomized controlled study showed that a four-day interactive training course on action-oriented home visits to pregnant women and young infants produced a sustained improvement of CHWs’ KAP and may represent a transferable model to ensure retention of acquired competencies and provide more solid foundations for improved CHWs performance.

## Additional files

Additional file 1: Questionnaire to assess knowledge, practices and attitudes of CHWs regarding home visits do pregnant women, mother and children. (DOCX 195 kb)

Additional file 2: **Table S1.** Pre-training Knowledge Assessment. Number of correct answers of CHWs before intervention. **Table S2.** Pre-training Attitudes Assessment. Number of correct answers (agree/totally agree with) of CHWs before intervention. **Table S3.** Pre-training Assessment of Practices. Number of correct answers (tasks often/always performed) of CHWs before intervention. **Table S4.** Knowledge assessment of intervention group (*n* = 31) pre-, post and one-year after training**. Table S5.** Knowledge assessment of intervention (*n* = 31) and control (*n* = 28) groups pre-and one-year after training. **Table S6.** Attitudes assessment of intervention group (n = 31) pre- and one-year after training. **Table S7.** Attitudes assessment of intervention (n = 31) and control (n = 28) groups pre-and one-year after training. **Table S8.** Practices assessment of intervention group (*n* = 31) pre- and one-year after training. **Table S9.** Practices assessment of intervention (n = 31) and control (n = 28) groups pre-and one-year after training. (DOCX 217 kb)

## Abbreviations

A: Attitudes
CHWs: Community Health Workers
FHTs: Family Health Teams
FHUs: Family Health Units
HDs: Heath Districts
HV: Home Visit
IMCI: Integrated Management of Childhood Illness
IMIP: Institute of Integral Medicine Professor Fernando Figueira
K: Knowledge
KAP: Knowledge, Attitudes and Practices
LMIC: Low and middle-income countries
MNCH: Maternal, Neonatal and Child Health
P: Practices
PACS: Program of Community Health Workers
PNAB: National Policy of Primary Care of Brazil
SPSS: Statistical Package for the Social Sciences
SUS: Sistema Único de Saúde (Brazilian Unified Public Health System)
t0: Pré-training
t1: Immediately after training
t2: One year after training
WHO: World Health Organization

## References

[1] Haines A, Sanders D, Lehmann U, Rowe AK, Lawn JE, Jan S (2007). Achieving child survival goals: potential contribution of community health workers. Lancet.

[2] Christopher JB, Le May A, Lewin S, Ross DA (2011). Thirty years after Alma-Ata: a systemic review of the impact of community health workers delivering curative interventions against malaria, pneumonia, and diarrhoea on child mortality and morbidity in sub-Saharan Africa. Hum Resour Health.

[3] Giugliani C, Harzheim E, Duncan MS, Duncan BB (2011). Effectiveness of community health Workers in Brazil: a systematic review. J Ambul Care Manage.

[4] Gilmore B, Mcauliffe E (2013). Effectiveness of community health workers delivering preventive interventions for maternal and child health in low- and middle-income countries: a systematic review. BMC Public Health.

[5] Lewin S, Munabi-Babigumira S, Glenton C, Daniels K, Bosch-Capblanch X, van Wyk BE, Odgaard-Jensen J, Johansen M, Aja GN, Zwarenstein M, Scheel IB. Lay health workers in primary and community health care for maternal and child health and the management of infectious diseases. Cochrane Database Syst Rev. 2010;17(3):CD004015. 10.1002/14651858.CD004015.pub3. 10.1002/14651858.CD004015.pub3 PMC6485809 20238326

[6] Gogia S, Sachdev HP (2016). Home-based neonatal care by community health workers for preventing mortality in neonates in low- and middle-income countries: a systematic review. J Perinatol.

[7] Lassi ZS, Middleton PF, Bhutta ZA (2016). Strategies for improving health care seeking for maternal and newborn illnesses in low- and middle-income countries: a systematic review and meta-analysis. Glob Health Action.

[8] Freeman P, Schleiff M, Sacks E, Rassekh B, Gupta S, Perry H (2017). Comprehensive review of the evidence regarding the effectiveness of community-based primary health care in improving maternal, neonatal and child health: 4. child health findings. J Glob Health.

[9] Jennings M, Pradhan S, Schleiff M, Sacks E, Freeman P, Gupta S (2017). Comprehensive review of the evidence regarding the effectiveness of community–based primary health care in improving maternal, neonatal and child health: 2. maternal health findings. J Glob Health.

[10] Sacks E, Freeman P, Sakyi K, Jennings M, Rassekh B, Gupta S (2017). Comprehensive review of the evidence regarding the effectiveness of community-based primary health care in improving maternal, neonatal and child health: 3. neonatal health findings. J Glob Health.

[11] Lehmann U, Sanders D (2007). Community health workers: what do we know about them? The state of the evidence on programmes, activities, costs and impact on health outcomes of using community health workers.

[12] Li VC, Goethals PR, Dorfman S (2006). A global review of training of community health workers. Int Q Community Health Educ.

[13] Jaskiewicz W, Tulenko K (2012). Increasing community health worker productivity and effectiveness: a review of the influence of the work environment. Hum Resour Health.

[14] Glenton C, Colvin CJ, Carlsen B, Swartz A, Lewin S, Noyes J, et al. Barriers and facilitators to the implementation of lay health worker programmes to improve access to maternal and child health: qualitative evidence synthesis. Cochrane Database Syst Rev. 2013;10:CD010414. 10.1002/14651858.CD010414.pub2. 10.1002/14651858.CD010414.pub2 PMC6396344 24101553

[15] Tran NT, Portela A, Bernis L, Beek K (2014). Developing capacities of community health workers in sexual and reproductive, maternal, newborn, child, and adolescent health: mapping and review of training resources. PLoS One.

[16] World Health Organization (1989). Strengthening the performance of community health workers. World Health Organization Technical Report.

[17] Brasil (2012). Política Nacional de Atenção Básica.

[18] Brasil (2001). Programa agentes comunitários de saúde (PACS).

[19] Brasil (2009). O trabalho do agente comunitário de Saúde.

[20] Drulla AG, Alexandre AMC, Rubel FI, Mazza VA (2009). A visita domiciliar como ferramenta ao cuidado familiar. Cogitare Enfermagem.

[21] Gaíva MAM, Siqueira VCA (2011). A prática da visita domiciliária pelos profissionais da Estratégia Saúde da Família. Cienc Cuid Saude.

[22] Zanchetta MS, McCrae Vander Voet S, Galhego-Garcia W, VMN S, Talbot Y, Riutort M, AMMF G, de Souza TJ, Caldas RS, Costa E, Kamikihara MM, Smolentzov S (2009). Effectiveness of community health agents’ actions in situations of social vulnerability. Health Educ Res.

[23] Gomes KO, Cotta RMM, Cherchiglia ML, Mitre SM, Batista RS (2009). A práxis do agente comunitário de saúde no contexto do programa saúde da família: reflexões estratégicas. Saude soc.

[24] Kebian LVA, Acioli S (2014). A visita domiciliar de enfermeiros e agentes comunitários de saúde da Estratégia Saúde da Família. Revista Eletrônica de Enfermagem.

[25] Braga GMAM, Mafra SCT, Silva EP, Gomes AP, Melo MSS (2016). Percepção do trabalho do agente comunitário de saúde pelos usuários atendidos nas unidades básicas de saúde da família de Viçosa, MG: tarefas realizadas e normas prescritas. Oikos: Revista Brasileira de Economia Doméstica.

[26] Ferraz L, Aerts DRGC (2005). O cotidiano de trabalho do agente comunitário de saúde no PSF em Porto Alegre. Cien Saude Colet.

[27] Albuquerque ABB, Bosi MLM (2009). Visita domiciliar no âmbito da Estratégia Saúde da Família: percepções de usuários no Município de Fortaleza, Ceará. Brasil Cad Saude Publica.

[28] Maciazeki-Gomes RC, Souza CD, Baggio L, Wachs F (2016). O trabalho do agente comunitário de saúde na perspectiva da educação popular em saúde: possibilidades e desafios. Ciência & Saúde Coletiva.

[29] Rotheram-Borus MJ, Tomlinson M, le Roux IM, Harwood JM, Comulada S (2014). A cluster randomised controlled effectiveness trial evaluating perinatal home visiting among south African mothers/infants. PLoS One.

[30] Tripathi A, Kabra SK, Sachdev HP, Lodha R (2016). Home visits by community health workers to improve identification of serious illness and care seeking in newborns and young infants from low- and middle-income countries. J Perinatol.

[31] Murray L, Cooper P, Arteche A, Stein A, Tomlinson M (2016). Randomized controlled trial of a home-visiting intervention on infant cognitive development in peri-urban South Africa. Dev Med Child Neurol.

[32] Brasil (2009). Guia prático do agente comunitário de saúde.

[33] Kirkpatrick D (1996). Great ideas revisited. Techniques for evaluating training programs. Revisiting Kirkpatrick’s four-level model. Training and Development.

[34] Dick W, Carey L, Carey JO, Dick W, Carey L, Carey JO (2015). Introduction to instructional design. The systematic design of instruction.

[35] Harden RM, Dent JA, Harden RM (2013). Curriculum planning and development. A practical guide for medical teachers.

[36] Hmelo-Silver CE (2004). Problem-based learning: what and how do students learn?. Educ psychol review.

[37] Moust JHC, Van Berkel HJM, Schmidt HG (2005). Signs of erosion: reflections on three decsdes of problem-based learning at Maastricht University. Higher Educ.

[38] Freire P (2006). Pedagogia da autonomia: saberes necessários à prática educativa.

[39] Mitre SM (2008). Metodologias ativas de ensino-aprendizagem na formação profissional em saúde: debates atuais. Ciência & Saúde Coletiva, Rio de Janeiro.

[40] Bordenave JD, Pereira AM (2002). Estratégias de ensino-aprendizagem.

[41] Tomaz JBC (2002). O agente comunitário de saúde não deve ser um “super-herói”. Interface - Comunic, Saude, Educ.

[42] Cyrino EG, Toralhes-Pereira ML (2004). Trabalhando com estratégias de ensino-aprendizado por descoberta na área de saúde: a problematização e a aprendizagem baseada em problemas. Cad Saúde Pública.

[43] Ribeiro BS, Carmo EA, Bomfim ES, Cardoso TSG, Duare ACS, Boery RNSO (2016). Metodologia da problematização no ensino em saúde: experiência com agentes comunitários de saúde. Revista de enfermagem UFPE.

[44] Lima TRM, Diniz PCMDM, Valente EP, Vezzini F, Tamburlini G (2017). Innovating home visiting to mothers and infants by community health workers: an action-oriented guide. Rev Bras Saúde Matern Infant.

[45] World Health Organization (2003). Integrated management of neonatal and childhood illnesses. WHO.

[46] King M, Bruner G (2000). Social desirability bias: a neglected aspect of validity testing. Psychol Mark.

[47] BornStein VJ, David HMSL, Araújo JWG (2010). Community health agents: reconstruction of the risk concept at local level. Interface - Comunic., Saude, Educ.

[48] Vidal SA, Silva EV, Oliveira MG, Siqueira AS, Felisberto E, Samico I (2003). Avaliação da aplicação da estratégia da atenção Integrada às doenças prevalentes da infância (AIDPI) por agentes comunitários de saúde. Rev Bras Saude Matern Infant.

[49] August F, Pembe AB, Mpembeni R, Axemo P, Darj E (2016). Effectiveness of the home based life saving skills training by community health workers on knowledge of danger signs, birth preparedness, complication readiness and facility delivery, among women in Rural Tanzania. BMC Pregnancy and Childbirth.

[50] Perry H, Rassekh B, Gupta S, Freeman P (2017). Comprehensive review of the evidence regarding the effectiveness of community-based primary health care in improving maternal, neonatal and child health: 6. strategies used by effective projects. J Glob Health.

[51] Duggan A, Windham A, McFarlane E, Fuddy L, Rohde C, Buchbinder S (2000). Hawaii’s healthy start program of home visiting for at-risk families: evaluation of family identification, family engagement, and service delivery. Pediatrics.

[52] Black MM, Walker SP, Fernald RCH (2017). Early childhood development coming of age: science through the life course. Lancet.

[53] Chan M (2013). Linking child survival and child development for health, equity, and sustainable development. Lancet.

[54] Martins CL, Oliveira SS, Rodrigues MA, Watanabe HAW, Jacomo YA (1996). Agentes comunitários nos serviços de saúde pública: elementos para uma discussão. Revista Saúde Debate.

[55] Silva JA, Dalmaso ASW (2002). Agente Comunitário de Saúde: o ser, o saber, o fazer.

[56] Santos KT, Saliba NA, Moimaz SAS, Arciere RM, Carvalho ML (2011). Agente comunitário de saúde: perfil adequado a realidade do Programa Saúde da Família?. Ciênc. saúde coletiva.

[57] Costa SM, Araújo FF, Martins LV, Nobre LLR, Araújo FM, Rodrigues CAQ (2013). Agente Comunitário de Saúde: elemento nuclear das ações em saúde. Ciênc saúde coletiva.

[58] Gagné RM, Briggs LJ, Wager WW, Gagné RM, Briggs LJ, Wager WW (1992). Designing of instructional systems. Principles of Instructional Design York: Holt, Rineheart & Winston.

[59] Posner GJ (1995). Concepts of curriculum and purposes of curriculum study. In: Posner GJ. Analyzing the curriculum.

[60] Shambaugh RN, Magliaro SG (1997). Mastering the possibilities; a process approach to instructional design.

[61] Cardoso FA, Cordeiro VRN, Lima DB, Melo BC, Menezes RNB, Moulaz ALS, Sá GB, Souza AVF (2011). Capacitação de agentes comunitários de saúde: experiência de ensino e prática com alunos de Enfermagem. Rev Bras Enferm.

[62] Pedrosa IL, Lira GA, Oliveira B, Silva MS, Santos MB, Silva EA (2011). Uso de metodologias ativas na formação técnica do agente comunitário de saúde. Trab Educ Saúde.

[63] World Health Organization (2012). Optimizing health worker roles to improve access to key maternal and newborn health interventions through task shifting.

