# Supplementary material for: Effectiveness of an action-oriented educational intervention in ensuring long term improvement of knowledge, attitudes and practices of community health workers in maternal and infant health: a randomized controlled study
Source: BMC Med Educ. 2018 Sep 27;18:224. doi: 10.1186/s12909-018-1332-x (PMC6161430; doi:10.1186/s12909-018-1332-x)
Supplement: Supplementary file 1 — Questionnaire to assess knowledge, practices and attitudes of CHWs regarding home visits do pregnant women, mother and children. (DOCX 195 kb) [file 12909_2018_1332_MOESM1_ESM.docx]

| **QUESTIONÁRIO DE AVALIAÇÃO DOS CONHECIMENTOS, ATITUDES E PRÁTICAS DOS PROFISSIONAIS DE SAÚDE DA ESTRATÉGIA DE SAÚDE DA FAMÍLIA (ESF) SOBRE SAÚDE MATERNO INFANTIL** | |
| --- | --- |
| **VERSÃO PARA AGENTES COMUNITÁRIOS DE SAÚDE (ACS)** | **Nº do Questionário:** |

*Prezado(a), este questionário tem como objetivo avaliar os conhecimentos, atitudes e práticas dos profissionais da ESF sobre saúde materno infantil. Por favor, leia cada questão cuidadosamente e responda de acordo com os enunciados. Todas as informações aqui registradas serão mantidas em confidencialidade. Sua colaboração e sinceridade nas respostas será muito importante para nós!*

| **PARTE I - IDENTIFICAÇÃO E PERFIL DOS PROFISSIONAIS** | |  |  |  |  |
| --- | --- | --- | --- | --- | --- |
|  |  | | | | |
| Distrito Sanitário: ( )1 ( )2 ( )4 | Nome (opcional):  ______________________________ | | | | |
| Nome da USF:_________________________________ | Idade:  ______ anos Gênero: ( ) M ( ) F | | | | |
| Número da equipe: ( )1 ( )2 | Tem filhos? ( ) Sim ( ) Não | | | | |
| Número da microárea: ( )1 ( )2 ( )4 ( )5 ( )6 | Bairro onde mora:______________________________ | | | | |
| Número de famílias na microárea: ______ famílias | Grau de escolaridade: ___________________________ | | | | |
| Tempo de serviço nesta unidade: _______ anos | Tempo de experiência na profissão: _______ anos | | | | |
|  | | | | | |
| 1.1. Você já fez algum curso ou especialização sobre temas relacionados à saúde da gestante? ( ) Sim ( ) Não | | | | | |
| Se sua resposta for sim, especifique o(s) tema(s):  _____________________________________________ ___________________________________________  _____________________________________________ ___________________________________________ | | | | | |
| Qual a duração do curso mais longo? _________ horas | | | | | |
|  | | | | | |
| 1.2. Você já fez algum curso ou especialização sobre temas relacionados à saúde da criança? ( ) Sim ( ) Não | | | | | |
| Se sua resposta for sim, especifique o(s) tema(s):  _____________________________________________ ___________________________________________  _____________________________________________ ___________________________________________ | | | | | |
| Qual a duração do curso mais longo? _________ horas | | | | | |
|  | | | | | |
| 1.3. Você já fez algum curso ou especialização sobre visitas domiciliares às gestantes e crianças? ( )Sim ( )Não | | | | | |
| Se sua resposta for sim, especifique o(s) tema(s):  _____________________________________________ ___________________________________________  _____________________________________________ ___________________________________________ | | | | | |
| Qual a duração do curso mais longo? _________ horas | | | | | |
|  | | | | | |
| 1.4. Você gostaria de participar de um treinamento específico sobre saúde materno infantil? ( )Sim ( )Não | | | | | |
| Se sua resposta for sim, quais os temas você tem interesse?  ( ) Saúde da gestante (pré-natal) ( ) Outros: ___________________________________  ( ) Saúde da puérpera (período puerperal) ___________________________________  ( ) Saúde do recém-nascido (período neonatal) ___________________________________  ( ) Saúde do criança – Alimentação ___________________________________  ( ) Saúde do criança – Crescimento e desenvolvimento ___________________________________  ( ) Saúde do criança – Vacinação ___________________________________  ( ) Saúde do criança – Prevenção de acidentes  ( ) Visitas domiciliares às gestantes e crianças | | | | | |
|  | | | | | |
| 1.5. Você tem/teve alguma dificuldade para participar de cursos de treinamento oferecidos? ( )Sim ( )Não | | | | | |
| Se sua resposta for sim, quais são as suas principais dificuldades?  ( ) Não tenho liberação da chefia/distrito ( ) Outros: ___________________________________  ( ) Os cursos são caros ___________________________________  ( ) Não tenho horários livres para participar ___________________________________  ( ) Não encontrei nenhum curso do meu interesse | | | | | |
| **PARTE II - CONHECIMENTOS** | | | | | |
|  | | | | | |
| 2.1. De acordo com o Ministério da saúde, qual o momento ideal para iniciar o pré-natal? | | | | | |
| a)     Antes da 12ª semana de gestação. | | | | | |
| b)     Da 12ª a 16ª semana de gestação. | | | | | |
| c)     Da 16ª a 20ª semana de gestação. | | | | | |
| d)     Após a 20ª semana de gestação. | | | | | |
| 2.2. Qual o número mínimo de consultas de pré-natal recomendado pelo Ministério da Saúde? | | | | | |
| a)     2 consultas. | | | | | |
| b)     4 consultas. | | | | | |
| c)     6 consultas. | | | | | |
| d)     8 consultas. | | | | | |
| 2.3. Segundo as recomendações atuais do Ministério da Saúde sobre o pré-natal, a gestante deve ser vacinada contra quais doenças? | | | | | |
| a)    Rubéola, Influenza e Sarampo. | | | | | |
| b)    Difteria, Coqueluche e Tétano. | | | | | |
| c)    Sarampo, Influenza e Tétano. | | | | | |
| d)   Tétano, Coqueluche e Rubéola. | | | | | |
| 2.4. Qual(is) da(s) situações descritas abaixo são consideradas fatores de risco durante a gravidez?  *(Nesta alternativa, você pode assinalar mais de uma resposta)* | | | | | |
| a)     Idade menor que 19 anos. f)     Ganho de peso excessivo.  b)     Dependência econômica. g)    Aumento do volume das mamas.  c)     Problemas conjugais. h)     Elevação da pressão arterial.  d)     Baixa escolaridade. i)     Enjoos no 1º trimestre.  e)     Gestação gemelar. j)      Aumento do sono. | | | | | |
| 2.5. Qual(is) da(s) situações descritas abaixo são consideradas sinais de alerta na gestante e devem ser encaminhadas ao médico ou enfermeiro?  *(Nesta alternativa, você pode assinalar mais de uma resposta)* | | | | | |
| a)    Náuseas e vômitos frequentes. f)      Sangramentos. | | | | | |
| b)     Corrimento vaginal com mau cheiro. g)     Edema nas mãos, pés e face. | | | | | |
| c)     Dor nas mamas. h)     Excesso de sono. | | | | | |
| d)     Aumento da frequência urinária. i)     Aumento na pigmentação da face. | | | | | |
| e)     Febre. j)     Ausência de movimentos fetais. | | | | | |
| 2.6. Segundo as recomendações atuais do Ministério da Saúde, quando deve ser feita a primeira visita domiciliar do recém-nascido após a alta da maternidade? | | | | | |
| a) Na primeira semana pós-parto. | | | | | |
| b)    Entre a primeira e segunda semana pós-parto. | | | | | |
| c)    Entre a segunda semana e um mês pós-parto. | | | | | |
| d)    Após o primeiro mês pós-parto. | | | | | |
| 2.7. Quando o bebê está em aleitamento materno exclusivo, qual a frequência das mamadas deve ser orientada? | | | | | |
| a) De 3 em 3 horas. | | | | | |
| b)    De 6 em 6 horas. | | | | | |
| c)    De 3 em 3 horas durante o dia e livre a noite. | | | | | |
| d)    Livre, sempre que o bebê quiser. | | | | | |
| 2.8. De acordo com os *10 passos para a alimentação saudável da criança com menos de 2 anos* recomendados pelo Ministério da Saúde, assinale a alternativa que contem uma orientação **correta**: | | | | | |
| a) Nos períodos mais quentes, deve-se oferecer água nos intervalos das mamadas. | | | | | |
| b)     A partir do sexto mês devem ser iniciados os sucos de frutas nos intervalos das mamadas. | | | | | |
| c)     Os alimentos complementares devem ser oferecidos liquidificados no copo ou colher. | | | | | |
| d)     A partir do sexto mês, devem ser oferecidos alimentos complementares e manter o leite materno. | | | | | |
| 2.9. Qual(is) da(s) situações descritas abaixo são consideradas fatores de risco para crianças?  *(Nesta alternativa, você pode assinalar mais de uma resposta)* | | | | | |
| a)    Peso ao nascer abaixo de 2500g. f)     Baixa escolaridade dos pais. | | | | | |
| b)     Mãe adolescente. g)    Problemas financeiros dos pais | | | | | |
| c)    Calendário vacinal atrasado. h)    Resfriados frequentes. | | | | | |
| d)    Febre após vacina. i)    Atraso no desenvolvimento. | | | | | |
| e)    Óbito de irmão com menos de 5 anos. j)     Desmame antes dos 6 meses. | | | | | |
| 2.10. Em uma visita domiciliar, encontra-se uma criança de 24 dias de vida com febre (38,5^o^C), porém ela está bem ativa e sem nenhum outro sintoma. Qual a conduta **mais adequada** nesse caso? | | | | | |
| a) Marcar consulta para o médico na mesma semana. | | | | | |
| b)     Orientar vigilância rigorosa e antitérmico, e reavaliar nos próximos 3 dias. | | | | | |
| c)     Encaminhar para um serviço de urgência. | | | | | |
| d)     Orientar meios físicos (banho e compressas) e tranquilizar a família pois não há outros sintomas. | | | | | |
| 2.11. Quais das doenças abaixo são prevenidas pelas vacinas incluídas no calendário vacinal da criança proposto pelo Ministério da Saúde? | | | | | |
| a) Tuberculose, leishmaniose e gripe. | | | | | |
| b)     Sarampo, tuberculose e difteria. | | | | | |
| c)     Meningite meningocócica, coqueluche e dengue. | | | | | |
| d)     Rubéola, hepatite A e esquistossomose. | | | | | |
| 2.12. Qual das situações abaixo devem ser **recomendadas** para prevenção de acidentes do lactente?  *(Nesta alternativa, você pode assinalar mais de uma resposta)* | | | | | |
| a)    Dormir na cama com os pais. | | | | | |
| b)     Dormir de barriga pra cima. | | | | | |
| c)    Dormir de barriga pra baixo. . | | | | | |
| d)     Brincar com animais domésticos. | | | | | |
| e)     Transportar em cadeirinhas específicas. | | | | | |
| 2.13. Em uma visita domiciliar, a mãe de uma criança de 1 ano solicita sua opinião sobre o uso do andador. Qual a orientação **mais adequada** neste caso? | | | | | |
| a)    Desaconselhar o uso do andador pelo risco de traumatismo craniano. | | | | | |
| b)     Aconselhar o uso do andador pelos seus  benefícios na prevenção de quedas. | | | | | |
| c)    Desaconselhar o uso do andador por prejuízo no desenvolvimento  neurológico. | | | | | |
| d)    Aconselhar o uso do andador pelo  benefício que traz à aquisição antecipada da marcha. | | | | | |
|  | | | | | |
| 2.14. Quais das formas abaixo é a **mais adequada** para avaliar o desenvolvimento de uma criança?   1. Perguntar aos pais e/ou cuidadores. 2. Colocar no gráfico dos marcos de desenvolvimento. 3. Comparar com outras crianças da mesma idade. 4. Não há, pois cada criança tem seu ritmo   2.15. Durante as visitas domiciliares, que práticas podem ser ensinadas pelo ACS para os pais e cuidadores para estimular o desenvolvimento neurológico, emocional e motor de crianças de até 2 anos?   1. ______________________________________________________________________________________ 2. ______________________________________________________________________________________ 3. ______________________________________________________________________________________ 4. ______________________________________________________________________________________ 5. ______________________________________________________________________________________ | | | | | |
|  | | | | | |
| 2.16. Durante as visitas domiciliares, que orientações devem ser dadas pelo ACS para os pais e cuidadores sobre como educar e disciplinar as crianças? | | | | | |
| 1. ______________________________________________________________________________________ 2. ______________________________________________________________________________________ 3. ______________________________________________________________________________________ 4. ______________________________________________________________________________________ 5. ______________________________________________________________________________________ | | | | | |
|  | | | | | |
| 2.17. Qual o período da vida é considerado o mais importante para o desenvolvimento cerebral de um indivíduo? | | | | | |
| a) Da concepção ao 2º ano de vida. | | | | | |
| b)    Do 2º ao 5º ano de vida. | | | | | |
| c)    Do 5º ao 10º ano de vida. | | | | | |
| d)    A adolescência. | | | | | |
| 2.18. Qual(is) da(s) prática(s) abaixo comprovadamente estimula(m) o desenvolvimento da criança desde o nascimento? *(Nesta alternativa, você pode assinalar mais de uma resposta)* | | | | | |
| a) Experiências musicais. | | | | | |
| b)     Massagem. | | | | | |
| c)     Assistir televisão. | | | | | |
| d)     Leitura em família. | | | | | |
| e)     Brincadeiras. | | | | | |
|  | | | | | |
| 2.19. Qual(is) dos fatore(s) abaixo influencia(m) **negativamente** o desenvolvimento de uma criança?  *(Nesta alternativa, você pode assinalar mais de uma resposta)* | | | | | |
| a) Pouco relacionamento social. | | | | | |
| b)    Violência doméstica. | | | | | |
| c)     Problema de saúde mental dos pais. | | | | | |
| d)     Tempo prolongado de uso de aparelhos digitais (televisão, celular, tablet, etc) | | | | | |
| e)     Baixo grau de escolaridade dos pais. | | | | | |
|  | | | | | |
| 2.20. Qual(is) do(s) resultado(s) abaixo pode(m) ser atingido(s) com as visitas domiciliares a gestantes e crianças?  *(Nesta alternativa, você pode assinalar mais de uma resposta)* | | | | | |
| a) Preparação da gestante para o parto e pós-parto. | | | | | |
| b)     Redução de partos prematuros e recém-nascidos de baixo peso. | | | | | |
| c)     Melhora do estado nutricional das crianças. | | | | | |
| d)     Maior desenvolvimento cognitivo e emocional. | | | | | |
| e)     Redução da violência infantil. | | | | | |

| **PARTE III - PRÁTICAS** |  |  |  |
| --- | --- | --- | --- |
| *Responda os itens a seguir com sinceridade, considerando o que você costuma fazer na sua prática.*  **SAÚDE DA GESTANTE** |  |  |  |
| 3.1. Como você faz para descobrir uma gestante na comunidade? |  |  |  |
| \| ( ) Espero que ela procure o serviço. \| \| --- \| \| ( ) Descubro pelo teste de gravidez na unidade. \| \| ( ) Descubro por conversas informais na comunidade. \| \| ( ) Pergunto nas minhas visitas domiciliares. \| |  |  |  |
| 3.2. Em média, quantas gestantes você visita o domicílio por mês? __________ |  |  |  |
| 3.3. Em média, quantas vezes você visita o domicílio de uma gestante durante cada período da gestação? |  |  |  |
| 1º trimestre: _____ 2º trimestre: _____ 3º trimestre: _____ Total: _____ |  |  |  |
| 3.4. Qual a duração média da sua visita domiciliar às gestantes? _______ minutos |  |  |  |

| *3.5. Leia as declarações abaixo e assinale de acordo com a frequência com a qual você desempenha as ações descritas nas suas visitas domiciliares às gestantes*  ***Use a seguinte legenda: (1) Nunca (2) Raramente (3) Às vezes (4) Frequentemente (5) Sempre*** | | | | | |
| --- | --- | --- | --- | --- | --- |
| - - 1. Eu verifico o preenchimento do cartão da gestante. | (1) | (2) | (3) | (4) | (5) |
| - - 1. Eu incentivo a leitura do cartão da gestante. | (1) | (2) | (3) | (4) | (5) |
| - - 1. Eu falo sobre a importância do pré-natal. | (1) | (2) | (3) | (4) | (5) |
| - - 1. Eu monitoro o acompanhamento pré-natal. | (1) | (2) | (3) | (4) | (5) |
| - - 1. Eu motivo as gestantes a comparecer às consultas de pré-natal. | (1) | (2) | (3) | (4) | (5) |
| - - 1. Eu ofereço informações sobre vacinação de rotina da gestante. | (1) | (2) | (3) | (4) | (5) |
| - - 1. Eu oriento sobre o uso do ácido fólico | (1) | (2) | (3) | (4) | (5) |
| - - 1. Eu faço orientações sobre alimentação da gestante. | (1) | (2) | (3) | (4) | (5) |
| - - 1. Eu faço prevenção do consumo de álcool e drogas na gestação. | (1) | (2) | (3) | (4) | (5) |
| - - 1. Eu oriento a gestante sobre atividade física na gestação. | (1) | (2) | (3) | (4) | (5) |
| - - 1. Eu falo sobre os benefícios do parto normal para a gestante e bebê. | (1) | (2) | (3) | (4) | (5) |
| - - 1. Eu falo sobre os benefícios do aleitamento materno. | (1) | (2) | (3) | (4) | (5) |
| - - 1. Eu falo sobre a importância do desenvolvimento infantil. | (1) | (2) | (3) | (4) | (5) |
| - - 1. Eu estimulo a gestante a cantar para o bebê na barriga. | (1) | (2) | (3) | (4) | (5) |
| - - 1. Eu estimulo a gestante a conversar com o bebê na barriga. | (1) | (2) | (3) | (4) | (5) |
| - - 1. Eu incentivo a participação do pai no pré-natal. | (1) | (2) | (3) | (4) | (5) |
| - - 1. Eu explico sobre sinais de alerta para buscar o serviço de saúde. | (1) | (2) | (3) | (4) | (5) |
| - - 1. Eu oriento sobre os sinais de trabalho de parto. | (1) | (2) | (3) | (4) | (5) |
| - - 1. Eu explico sobre sinais de alerta na gravidez. | (1) | (2) | (3) | (4) | (5) |
| - - 1. Eu identifico gestantes de risco. | (1) | (2) | (3) | (4) | (5) |
| - - 1. Eu encaminho as gestantes com problemas para consultas na unidade. | (1) | (2) | (3) | (4) | (5) |
| - - 1. Eu falo sobre a importância dos exames de pré-natal. | (1) | (2) | (3) | (4) | (5) |
| - - 1. Eu ofereço explicações sobre o parto e trabalho de parto. | (1) | (2) | (3) | (4) | (5) |
| - - 1. Eu ofereço explicações sobre o pós-parto. | (1) | (2) | (3) | (4) | (5) |
| - - 1. Eu oriento sobre contracepção futura e planejamento familiar. | (1) | (2) | (3) | (4) | (5) |

3.6. Além das ações acima, descreva outra ações que você desempenha nas visitas domiciliares às gestantes:

1. ______________________________________________________________________________________
2. ______________________________________________________________________________________
3. ______________________________________________________________________________________
4. ______________________________________________________________________________________
5. ______________________________________________________________________________________

| 3.7. . São realizadas atividades educativas/grupos para as gestantes da unidade? ( ) Sim ( )Não |
| --- |
| Se sua resposta for sim, responda as perguntas abaixo:  3.7.1. Quais são os temas abordados? ____________________________________________________________  ___________________________________________________________________________________________  3.7.2. Qual a frequência das atividades? ( )diária ( )semanal ( )mensal ( ) outra: _____________  3.7.3. Quais os profissionais responsáveis por essas atividades? _______________________________________  ___________________________________________________________________________________________  3.7.4. Além das gestantes, quem participa dessas atividades? _________________________________________  ___________________________________________________________________________________________  3.7.5. Quais os recursos e materiais educativos são utilizados nessas atividades? _________________________  ___________________________________________________________________________________________ |

| **SAÚDE DA CRIANÇA** |
| --- |
| \| 3.8. Como você faz para saber que uma mulher que acabou de ter um filho já voltou à comunidade? \| \| --- \| \| ( ) Espero que ela procure o serviço. \| \| ( ) Peço para a família avisar. \| \| ( ) Entro em contato com a maternidade. \| \| ( ) Pergunto nas minhas visitas domiciliares. \| \| 3.9. Em média, qual a idade do bebê na sua primeira visita domiciliar após o nascimento? \| \| ( ) até 3 dias ( ) 4 a 7 dias ( ) 8 a 15 dias ( ) 16 a 30 dias ( ) mais de 30 dias  3.10. Em média, quantas crianças abaixo de 1 ano você visita o domicílio por mês?_________ \| \| 3.11. Quantas vezes você visita o domicílio de uma criança da sua microárea durante os primeiros 9 meses?  0 a 2 meses: _____ 2 a 3 meses: ____ 5 a 6 meses: _____ 8 a 9 meses: _____ Total: _____  3.12. Qual a duração média da sua visita domiciliar a uma criança de até 1 ano? ________ minutos \| |

| *3.13. Leia as declarações abaixo e assinale de acordo com a frequência com a qual você desempenha as ações descritas nas suas visitas domiciliares a crianças*  ***Use a seguinte legenda: (1) Nunca (2) Raramente (3) Às vezes (4) Frequentemente (5) Sempre*** | | | | | |
| --- | --- | --- | --- | --- | --- |
| - - 1. Eu verifico a caderneta da criança. | (1) | (2) | (3) | (4) | (5) |
| - - 1. Eu pergunto à mãe/cuidador se há algum problema com a criança. | (1) | (2) | (3) | (4) | (5) |
| - - 1. Eu peso a criança. | (1) | (2) | (3) | (4) | (5) |
| - - 1. Eu registro o peso no gráfico da caderneta. | (1) | (2) | (3) | (4) | (5) |
| - - 1. Eu meço o comprimento/estatura da criança. | (1) | (2) | (3) | (4) | (5) |
| - - 1. Eu registro o comprimento/estatura no gráfico da caderneta. | (1) | (2) | (3) | (4) | (5) |
| - - 1. Eu pergunto à mãe sobre o desenvolvimento da criança. | (1) | (2) | (3) | (4) | (5) |
| - - 1. Eu observo e avalio o desenvolvimento da criança. | (1) | (2) | (3) | (4) | (5) |
| - - 1. Eu registro os marcos do desenvolvimento na caderneta da criança. | (1) | (2) | (3) | (4) | (5) |
| - - 1. Eu oriento a mãe sobre o uso da caderneta da criança. | (1) | (2) | (3) | (4) | (5) |
| - - 1. Eu falo sobre a importância do desenvolvimento infantil. | (1) | (2) | (3) | (4) | (5) |
| - - 1. Eu explico a mãe/cuidador sobre os marcos do desenvolvimento. | (1) | (2) | (3) | (4) | (5) |
| - - 1. Eu estimulo a mãe/cuidador a conversar com a criança. | (1) | (2) | (3) | (4) | (5) |
| - - 1. Eu estimulo a mãe/cuidador a ler para criança. | (1) | (2) | (3) | (4) | (5) |
| - - 1. Eu explico a mãe/cuidador sobre a importância da leitura para a criança | (1) | (2) | (3) | (4) | (5) |
| - - 1. Eu estimulo a mãe/cuidador a contar histórias para a criança. | (1) | (2) | (3) | (4) | (5) |
| - - 1. Eu estimulo a mãe/cuidador a brincar com a criança. | (1) | (2) | (3) | (4) | (5) |
| - - 1. Eu estimulo a mãe/cuidador a mostrar objetos coloridos para a criança. | (1) | (2) | (3) | (4) | (5) |
| ***Legenda: (1) Nunca (2) Raramente (3) Às vezes (4) Frequentemente (5) Sempre*** | | | | | |
| - - 1. Eu estimulo a mãe/cuidador a ouvir música e cantar com a criança. | (1) | (2) | (3) | (4) | (5) |
| - - 1. Eu estimulo a usar momentos de rotina como momento de estímulos. | (1) | (2) | (3) | (4) | (5) |
| - - 1. Eu incentivo a participação do pai nos cuidados da criança. | (1) | (2) | (3) | (4) | (5) |
| - - 1. Eu observo a relação da criança com os pais/cuidadores. | (1) | (2) | (3) | (4) | (5) |
| - - 1. Eu falo sobre os benefícios do aleitamento materno. | (1) | (2) | (3) | (4) | (5) |
| - - 1. Eu oriento sobre a pega e posição corretas para a amamentação. | (1) | (2) | (3) | (4) | (5) |
| - - 1. Eu verifico a mamada e corrijo erros. | (1) | (2) | (3) | (4) | (5) |
| - - 1. Eu falo sobre os cuidados com a mama durante a amamentação. | (1) | (2) | (3) | (4) | (5) |
| - - 1. Eu oriento sobre o desmame e armazenamento de leite materno. | (1) | (2) | (3) | (4) | (5) |
| - - 1. Eu falo sobre riscos do uso de mamadeiras, chupetas e bicos artificiais. | (1) | (2) | (3) | (4) | (5) |
| - - 1. Eu faço orientações gerais sobre a alimentação da criança. | (1) | (2) | (3) | (4) | (5) |
| - - 1. Eu oriento sobre quantidade e qualidade dos alimentos para a criança. | (1) | (2) | (3) | (4) | (5) |
| - - 1. Eu oriento sobre os horários das refeições das crianças. | (1) | (2) | (3) | (4) | (5) |
| - - 1. Eu faço orientações sobre as vacinas e o calendário vacinal. | (1) | (2) | (3) | (4) | (5) |
| - - 1. Eu oriento sobre hábitos de higiene da criança. | (1) | (2) | (3) | (4) | (5) |
| - - 1. Eu oriento sobre higiene bucal da criança. | (1) | (2) | (3) | (4) | (5) |
| - - 1. Eu oriento sobre prevenção de acidentes. | (1) | (2) | (3) | (4) | (5) |
| - - 1. Eu oriento sobre sinais de alerta para levar a criança a serviço de saúde. | (1) | (2) | (3) | (4) | (5) |
| - - 1. Eu busco sinais de que a criança esteja em risco. | (1) | (2) | (3) | (4) | (5) |
| - - 1. Eu busco sinais de que a criança esteja sofrendo violência. | (1) | (2) | (3) | (4) | (5) |
| - - 1. Eu identifico problemas no crescimento e desenvolvimento. | (1) | (2) | (3) | (4) | (5) |
| - - 1. Eu identifico problemas na amamentação. | (1) | (2) | (3) | (4) | (5) |

3.14. Além das ações acima, descreva outra ações que você desempenha nas visitas domiciliares a crianças:

1. ______________________________________________________________________________________
2. ______________________________________________________________________________________
3. ______________________________________________________________________________________
4. ______________________________________________________________________________________
5. ______________________________________________________________________________________

| 3.15. São realizadas atividades educativas para pais e cuidadores sobre saúde da criança? ( ) Sim ( )Não |
| --- |
| Se sua resposta for sim, responda as perguntas abaixo:  3.15.1. Quais são os temas abordados? ___________________________________________________________  ___________________________________________________________________________________________  3.15.2. Qual a frequência das atividades? ( )diária ( )semanal ( )mensal ( ) outra: ____________  3.15.3. Quais os profissionais responsáveis por essas atividades? ______________________________________  ___________________________________________________________________________________________  3.15.4. Quem participa dessas atividades? ________________________________________________________  ___________________________________________________________________________________________  3.15.5. Quais os recursos e materiais educativos são utilizados nessas atividades? _________________________  ___________________________________________________________________________________________ |
| 3.16. Qual(is) sua(s) conduta(s) quando identifica problemas no crescimento/desenvolvimento de uma criança? |
| ( ) Faz orientações sobre alimentação. |
| ( ) Faz orientações sobre estímulo ao desenvolvimento. |
| ( ) Aumenta a frequência das visitas. |
| ( ) Marca consulta com enfermeiro/médico.  ( ) Outras: _____________________________________ |
| 3.17. Qual(is) a(s) sua(s) conduta(s) quando identifica problemas na amamentação de uma criança? |
| ( ) Faz orientações sobre aleitamento materno. |
| ( ) Aumenta a frequência das visitas. |
| ( ) Marca consulta com enfermeiro/médico.  ( ) Outras: _____________________________________ |
| 3.18. Qual(is) a(s) sua(s) conduta(s) quando identifica atraso vacinal durante a visita a uma criança? |
| ( ) Encaminho no mesmo dia para atualização. |
| ( ) Aguardo a próxima consulta para atualização. |
| ( ) Aguardo a próxima campanha de vacinação para atualização.  ( ) Outras: _____________________________________ |

| **PARTE IV - ATITUDES** |
| --- |

| *4.1. Leia as alternativas abaixo e assinale de acordo com grau de concordância com as declarações descritas.*  ***Use a seguinte legenda: (1) Discordo totalmente (2) Discordo parcialmente (3) Nem concordo nem discordo (4) Concordo parcialmente (5) Concordo totalmente*** | | | | | |
| --- | --- | --- | --- | --- | --- |
| - - 1. Eu sou pontual e assíduo nas minhas atividades da unidade. | (1) | (2) | (3) | (4) | (5) |
| - - 1. Eu desenvolvo adequadamente as minhas atividades de trabalho. | (1) | (2) | (3) | (4) | (5) |
| - - 1. Eu me comunico adequadamente com os usuários. | (1) | (2) | (3) | (4) | (5) |
| - - 1. Eu uso linguagem clara e simples para me comunicar com os usuários. | (1) | (2) | (3) | (4) | (5) |
| - - 1. Eu ouço com paciência as situações trazidas pelas famílias. | (1) | (2) | (3) | (4) | (5) |
| - - 1. Eu respeito as opiniões, crenças, e religião dos usuário. | (1) | (2) | (3) | (4) | (5) |
| - - 1. Eu me relaciono bem com os usuários. | (1) | (2) | (3) | (4) | (5) |
| - - 1. Eu me preocupo com a saúde dos usuários. | (1) | (2) | (3) | (4) | (5) |
| - - 1. Eu busco atualizar meus conhecimentos sobre saúde da gestante. | (1) | (2) | (3) | (4) | (5) |
| - - 1. Eu me considero capacitado para dar assistência a gestantes no domicílio | (1) | (2) | (3) | (4) | (5) |
| - - 1. Eu busco atualizar meus conhecimentos sobre saúde da criança. | (1) | (2) | (3) | (4) | (5) |
| - - 1. Eu me considero capacitado para dar assistência a crianças no domicílio. | (1) | (2) | (3) | (4) | (5) |
| - - 1. Eu acredito na importância do cartão da gestante e caderneta da criança. | (1) | (2) | (3) | (4) | (5) |
| - - 1. Quando eu tenho dúvidas, busco apoio dos outros membros da equipe. | (1) | (2) | (3) | (4) | (5) |
| - - 1. Eu me relaciono adequadamente com os outros profissionais da equipe. | (1) | (2) | (3) | (4) | (5) |
| - - 1. Eu analiso e acolho as críticas ou sugestões recebidas. | (1) | (2) | (3) | (4) | (5) |
| - - 1. Eu tenho refletido sobre o meu processo de trabalho na unidade. | (1) | (2) | (3) | (4) | (5) |
| - - 1. Eu conheço os indicadores de saúde da população que acompanho | (1) | (2) | (3) | (4) | (5) |
| - - 1. Eu me sinto feliz e satisfeito com o trabalho que tenho feito. | (1) | (2) | (3) | (4) | (5) |
| - - 1. Eu percebo resultados satisfatórios do meu trabalho. | (1) | (2) | (3) | (4) | (5) |

Caso tenha mais algo que queira acrescentar, use o espaço abaixo.

_________________________________________________________________________________________________________________________________________________________________________________________________________________________________________________________________________________________________________________________________________________________________________________________________________________________________________________________________________________

*Agradecemos a sua colaboração!*
