# Supplementary material for: Effectiveness of an action-oriented educational intervention in ensuring long term improvement of knowledge, attitudes and practices of community health workers in maternal and infant health: a randomized controlled study
Source: BMC Med Educ. 2018 Sep 27;18:224. doi: 10.1186/s12909-018-1332-x (PMC6161430; doi:10.1186/s12909-018-1332-x)
Supplement: Supplementary file 2 — Table S1. Pre-training Knowledge Assessment. Number of correct answers of CHWs before intervention. Table S2. Pre-training Attitudes Assessment. Number of correct answers (agree/totally agree with) of CHWs before intervention. Table S3. Pre-training Assessment of Practices. Number of correct answers (tasks often/always performed) of CHWs before intervention. Table S4. Knowledge assessment of intervention group (n = 31) pre-, post and one-year after training. Table S5. Knowledge assessment of intervention (n = 31) and control (n = 28) groups pre-and one-year after training. Table S6. Attitudes assessment of intervention group (n = 31) pre- and one-year after training. Table S7. Attitudes assessment of intervention (n = 31) and control (n = 28) groups pre-and one-year after training. Table S8. Practices assessment of intervention group (n = 31) pre- and one-year after training. Table S9. Practices assessment of intervention (n = 31) and control (n = 28) groups pre-and one-year after training. (DOCX 217 kb) [file 12909_2018_1332_MOESM2_ESM.docx]

**Additional file 2 – Supplementary tables**

| **Supplementary Table 1** Pre-training Knowledge Assessment. Number of correct answers of CHWs before intervention. | | | | | | | | |  |
| --- | --- | --- | --- | --- | --- | --- | --- | --- | --- |
|  |  | Groups | | | | |  | |  |
| Correct answers | Total (n=59) | Intervention (n=31) | | | | Control (n=28) | p-value | |  |
|  | n (%) | n (%) | | | | n (%) |  |  |  |
| Risk factors for pregnant women |  |  | | | |  |  | |  |
| Age under 19 years | 47 (79.7) | 25 (80.6) | | | | 22 (78.6) | 0.843 | |  |
| Economic dependence | 2 (3.4) | 1 (3.2) | | | | 1 (3.6) | 1.000 | |  |
| Marital problems | 7 (11.9) | 3 (9.7) | | | | 4 (14.3) | 0.698 | |  |
| Low educational level | 8 (13.6) | 3 (9.7) | | | | 5 (17.9) | 0.458 | |  |
| Twin pregnancy | 31 (52.5) | 15 (48.4) | | | | 16 (57.1) | 0.501 | |  |
| Excessive weight gain | 48 (81.4) | 26 (83.9) | | | | 22 (78.6) | 0.602 | |  |
| Blood pressure elevation | 55 (93.2) | 28 (90.3) | | | | 27 (96.4) | 0.614 | |  |
| Warning signs in pregnant women |  |  | | | |  |  | |  |
| Frequent nausea and vomiting | 15 (25.4) | 8 (25.8) | | | | 7 (25.0) | 0.943 | |  |
| Vaginal discharge with bad smell | 51 (86.4) | 26 (83.9) | | | | 25 (89.3) | 0.709 | |  |
| Fever | 48 (81.4) | 25 (80.6) | | | | 23 (82.1) | 0.883 | |  |
| Bleeding | 55 (93.2) | 27 (87.1) | | | | 28 (100.0) | 0.114 | |  |
| Edema on hands. feet and face | 42 (71.2) | 21 (67.7) | | | | 21 (75.0) | 0.539 | |  |
| Absence of fetal movements | 50 (84.7) | 25 (80.6) | | | | 25 (89.3) | 0.477 | |  |
| Risk factors/warning signs for child |  |  | | | |  |  | |  |
| Birth weight below 2500g | 56 (94.9) | 28 (90.3) | | | | 28 (100.0) | 0.239 | |  |
| Teen Mother | 40 (67.8) | 24 (77.4) | | | | 16 (57.1) | 0.096 | |  |
| Late vaccination schedule | 49 (83.1) | 28 (90.3) | | | | 21 (75.0) | 0.168 | |  |
| Death of a under 5 year sibling | 8 (13.6) | 3 (9.7) | | | | 5 (17.9) | 0.458 | |  |
| Low parental education | 8 (13.6) | 4 (12.9) | | | | 4 (14.3) | 1.000 | |  |
| Parents' financial problems | 3 (5.1) | 1 (3.2) | | | | 2 (7.1) | 0.599 | |  |
| Delay in development | 37 (62.7) | 22 (71.0) | | | | 15 (53.6) | 0.168 | |  |
| Early weaning | 37 (62.7) | 18 (58.1) | | | | 19 (67.9) | 0.437 | |  |
| Antenatal care |  |  | | | |  |  | |  |
| Ideal period to start prenatal care | 53 (93.0) | 28 (93.3) | | | | 25 (92.6) | 1.000 | |  |
| Number of prenatal consultations | 38 (64.4) | 19 (61.3) | | | | 19 (67.9) | 0.888 | |  |
| Vaccination in pregnancy | 27 (48.2) | 15 (50.0) | | | | 12 (46.2) | 0.142 | |  |
| Newborn care |  |  | | | |  |  | |  |
| Moment of first home visit | 45 (73.6) | 22 (71.0) | | | | 23 (82.1) | 0.805 | |  |
| Frequency of breastfeeding | 46 (78.0) | 26 (83.9) | | | | 20 (71.4) | 0.197 | |  |
| Care for newborn with fever | 38 (64.4) | 17 (54.8) | | | | 21 (75.0) | 0.493 | |  |
| Child care |  |  | | | |  |  | |  |
| Nutrition in the first 2 years old | 45 (76.3) | 25 (80.6) | | | | 20 (71.4) | 0.467 | |  |
| Vaccination in children | 56 (94.9) | 29 (93.5) | | | | 27 (96.4) | 1.000 | |  |
| Prevention of accidents |  |  | | | |  |  | |  |
| - Sleeping position | 33 (55.9) | 20 (64.5) | | | | 13 (46.4) | 0.163 | |  |
| - Use of infant seats | 42 (71.2) | 25 (80.6) | | | | 17 (60.7) | 0.077 | |  |
| - Risks of child walker | 36 (62.1) | 19 (61.3) | | | | 17 (63.0) | 0.216 | |  |
| Child development |  |  | | | |  |  | |  |
| Assessment of child development | 42 (71.2) | 22 (71.0) | | | | 20 (71.4) | 0.277 | |  |
| Important stages for brain development | 40 (70.2) | 24 (80.0) | | | | 16 (59.3) | 0.156 | |  |
| Practices that stimulate child development |  |  | | | |  |  | |  |
| - Musical Experiences | 43 (74.1) | 22 (73.3) | | | | 21 (75.0) | 0.885 | |  |
| - Massage | 32 (55.1) | 17 (56.7) | | | | 15 (53.6) | 0.813 | |  |
| - Family Reading | 42 (72.4) | 23 (76.7) | | | | 19 (67.9) | 0.453 | |  |
| - Playing | 44 (75.9) | 24 (80.0) | | | | 20 (71.4) | 0.446 | |  |
| Risk factors for child development |  |  | | | |  |  | |  |
| - Little social relationship | 45 (77.6) | 24 (80.0) | | | | 21 (75.0) | 0.648 | |  |
| - Domestic Violence | 54 (93.1) | 30 (100.0) | | | | 24 (85.7) | **0.048** | |  |
| - Parents' mental health problem | 31 (53.4) | 19 (63.3) | | | | 12 (42.9) | 0.118 | |  |
| - Extended use of digital devices | 27 (46.6) | 15 (50.0) | | | | 12 (42.9) | 0.586 | |  |
| - Parents' low level of education | 14 (24.1) | | 8 (26.7) | | | 6 (21.4) | 0.641 | | |
| Aims of home visits to mother and child health |  | |  |  | | | | | |
| - Preparing for childbirth and postpartum | 45 (77.6) | | 23 (76.7) | | 22 (78.6) | | | 0.862 | |
| - Reduction of preterm and low birth weight | 27 (46.6) | | 18 (60.0) | | 9 (32.1) | | | **0.034** | |
| - Improvement of children's nutritional status | 50 (86.2) | | 28 (93.3) | | 22 (78.6) | | | 0.138 | |
| - Greater cognitive and emotional development | 29 (50.0) | | 16 (53.3) | | 13 (46.4) | | | 0.599 | |
| - Reduction of child violence | 33 (56.9) | | 20 (66.7) | | 13 (46.4) | | | 0.120 | |

| **Supplementary Table 2** Pre-training Attitudes Assessment. Number of correct answers (agree/totally agree with) of CHWs before intervention | | | | |
| --- | --- | --- | --- | --- |
|  |  | Groups | |  |
| Behaviors | Total (n=59) | Intervention (n=31) | Control (n=28) | p-value |
|  | n (%) | n (%) | n (%) |  |
| Is punctual and assiduous in work activities. | 53 (89.8) | 29 (96.7) | 24 (88.9) | 0.336 |
| Perform work activities properly. | 53 (89.8) | 28 (96.6) | 25 (92.6) | 0.605 |
| Communicate appropriately with users. | 57 (96.6) | 31 (100.0) | 26 (96.3) | 0.466 |
| Use clear and simple language to communicate with users. | 59 (100.0) | 31 (100.0) | 28 (100.0) | na |
| Listen patiently to the situations brought by families. | 57 (96.6) | 31 (100.0) | 26 (96.3) | 0.466 |
| Respect the opinions, beliefs and religion of the users. | 57 (96.6) | 31 (100.0) | 26 (96.3) | 0.466 |
| Have good relationship with users. | 56 (94.9) | 31 (100.0) | 25 (92.6) | 0.220 |
| Have concerns for the health of users. | 57 (96.6) | 31 (100.0) | 26 (96.3) | 0.466 |
| Seek to update the knowledge about pregnant health. | 53 (89.8) | 30 (96.8) | 23 (85.2) | 0.173 |
| Is capable to provide assistance to pregnant women at home | 50 (84.7) | 27 (87.1) | 23 (85.2) | 1.000 |
| Seek to update the knowledge about child health. | 59 (100.0) | 31 (100.0) | 28 (100.0) | na |
| Is capable to provide assistance to children at home. | 51 (86.4) | 29 (93.5) | 22 (85.1) | 0.233 |
| Consider the importance of the cards of pregnant and child. | 59 (100.0) | 31 (100.0) | 28 (100.0) | na |
| When in doubt, seek support from other team members. | 59 (100.0) | 31 (100.0) | 28 (100.0) | na |
| Has good relationship with the other team members. | 57 (96.6) | 31 (100.0) | 26 (96.3) | 0.466 |
| Analyze and receive critics or suggestions. | 50 (84.7) | 28 (96.6) | 22 (85.1) | 0.096 |
| Reflects on your work process. | 54 (91.5) | 29 (96.7) | 25 (92.6) | 0.599 |
| Know the health indicators of the population that assist. | 48 (81.3) | 28 (96.6) | 20 (74.1) | **0.023** |
| Feel happy and satisfied her/his your work. | 46 (77.9) | 28 (90.3) | 19 (70.4) | 0.053 |
| Perceive satisfactory results of your work. | 55 (93.2) | 29 (93.5) | 26 (96.3) | 1.000 |

na not available. p-value could not be calculated due to 100 % correct answers.

| **Supplementary Table 3** Pre-training Assessment of Practices. Number of correct answers (tasks often/always performed) of CHWs before intervention. | | | | | | | |
| --- | --- | --- | --- | --- | --- | --- | --- |
|  |  | Groups | | | |  | |
| Tasks | Total (n=59) | Intervention (n=31) | | Control (n=28) | | p-value | |
|  | n (%) | n (%) | | n (%) | |  |  |
| Task performed at home visits to pregnant women |  |  | |  | |  | |
| Identify pregnant women at risk. | 45 (76.2) | 23 (76.7) | | 22 (81.5) | | 0.656 | |
| Identify warning signs in pregnancy. | 51 (86.4) | 27 (90.0) | | 24 (85.7) | | 0.701 | |
| Explain about warning signs to get health care. | 54 (91,5) | 29 (96.7) | | 25 (89.3) | | 0.344 | |
| Forward pregnant women with problems for the unit. | 59 (100.0) | 31 (100.0) | | 28 (100.0) | | na | |
| Talk about the importance of prenatal care. | 57 (96.6) | 31 (100.0) | | 26 (92.9) | | 0.221 | |
| Motivate pregnant women to attend consultations. | 59 (100.0) | 31 (100.0) | | 28 (100.0) | | na | |
| Encourage the father's participation in prenatal care. | 31 (52.5) | 16 (53.3) | | 15 (53.6) | | 0.986 | |
| Monitor the prenatal follow-up. | 58 (98.3) | 30 (96.8) | | 28 (100.0) | | 1.000 | |
| Verify the fill of the pregnant woman's card. | 44 (74.5) | 23 (76.7) | | 21 (75.0) | | 0.882 | |
| Encourage the reading of the pregnant woman's card. | 25 (42.3) | 12 (41.4) | | 13 (46.4) | | 0.701 | |
| Talk about the importance of prenatal exams. | 55 (93.2) | 28 (90.3) | | 27 (96.4) | | 0.614 | |
| Inform about vaccination schedule in pregnancy | 55 (93.2) | 28 (90.3) | | 27 (96.4) | | 0.614 | |
| Inform about correct nutrition in pregnancy | 44 (74.5) | 25 (80.6) | | 19 (73.1) | | 0.498 | |
| Inform about iron replacement | 38 (64,4) | 20 (66.7) | | 18 (66.7) | | 1.000 | |
| Orient about physical activity in pregnancy. | 21 (35.6) | 11 (36.7) | | 10 (37.0) | | 0.977 | |
| Alert on alcohol and drug risks in pregnancy. | 43 (72.8) | 22 (71.0) | | 21 (75.0) | | 0.728 | |
| Explain about child development in pregnancy | 49 (83.0) | 23 (79.3) | | 26 (92.9) | | 0.253 | |
| Stimulate mother to sing for the baby in the belly. | 20 (33.9) | 11 (37.9) | | 9 (36.0) | | 0.884 | |
| Stimulate the mother to talk to the baby in the belly. | 37 (62.7) | 21 (67.7) | | 16 (57.1) | | 0.401 | |
| Talk about the benefits of normal birth. | 49 (83.0) | 25 (86.0) | | 24 (85.7) | | 0.734 | |
| Explain about the signs of labor. | 46 (77.9) | 23 (74.2) | | 23 (82.1) | | 0.462 | |
| Explain about labor and childbirth. | 31 (52.5) | 16 (53.3) | | 15 (53.6) | | 0.986 | |
| Provide explanations about postpartum. | 39 (66.1) | 19 (63.3) | | 20 (71.4) | | 0.512 | |
| Talk about the benefits of breastfeeding. | 57 (96.6) | 29 (93.5) | | 28 (100.0) | | 0.493 | |
| Orient about future contraception and family planning. | 48 (81.3) | 22 (73.3) | | 26 (92.9) | | 0.081 | |
| Tasks performed at home visits to mother and child. |  |  | |  | |  | |
| Identify children at risk. | 42 (71.1) | 21 (72.4) | | 21 (75.0) | | 0.825 | |
| Identify signs of child suffering violence. | 41 (69.5) | 21 (72.4) | | 20 (71.4) | | 0.934 | |
| Observe relationship of child and parents/caregivers. | 47 (79.6) | 23 (79.3) | | 24 (85.7) | | 0.730 | |
| Encourage the father's involvement in childcare. | 42 (71.1) | 22 (75.9) | | 21 (75.0) | | 0.940 | |
| Ask mother/caregiver about problem with the child. | 59 (100.0) | 31 (100.0) | | 28 (100.0) | | na | |
| Explain about warning signs to get child to health care. | 54 (91.5) | 29 (100.0) | | 25 (96.2) | | 0.473 | |
| Verify the fill of the child's card. | 57 (96.6) | 31 (100.0) | | 26 (92.9) | | 0.221 | |
| Instruct mother/caregiver about the child's card. | 51 (86.4) | 27 (93.1) | | 24 (88.9) | | 0.664 | |
| Inform about child vaccination schedule. | 56 (94.9) | 30 (100.0) | | 26 (96.3) | | 0.474 | |
| Talk about the benefits of breastfeeding. | 56 (94.9) | 28 (96.6) | | 28 (100.0) | | 1.000 | |
| Orient about breastfeeding position and latch-on. | 55 (93.2) | 28 (93.3) | | 27 (96.4) | | 1.000 | |
| Observe attachment and correct errors. | 52 (88.1) | 27 (90.0) | | 25 (92.6) | | 1.000 | |
| Identify problems in breastfeeding. | 49 (83.0) | 25 (86.2) | | 24 (85.7) | | 1.000 | |
| Talk about breast care during breastfeeding. | 54 (91.5) | 29 (96.7) | | 25 (92.6) | | 0.599 | |
| Orient about weaning and storage of breast milk. | 40 (67.8) | 19 (63.6) | | 21 (75.0) | | 0.337 | |
| Alert on risks of baby bottles and pacifiers. | 56 (94.9) | 28 (93.3) | | 28 (100.0) | | 0.492 | |
| Inform about correct child nutrition. | 47 (79.7) | 24 (82.8) | | 23 (88.5) | | 0.708 | |
| Inform about quantity and quality of child food. | 51 (86.4) | 26 (89.7) | | 25 (92.6) | | 1.000 | |
| Orient about child meal times. | 46 (77.9) | 23 (85.2) | | 23 (85.2) | | 1.000 | |
| Explain about correct child hygiene habits. | 55 (93.2) | 28 (96.6) | | 27 (100.0) | | 1.000 | |
| Explain about oral hygiene of the child. | 52 (88.1) | 27 (93.1) | | 25 (92.6) | | 1.000 | |
| Inform about accident prevention. | 50 (84.7) | 26 (89.7) | | 24 (88.9) | | 1.000 | |
| Ask mother/caregiver about the child's development. | 43 (72.8) | 24 (85.7) | | 19 (73.1) | | 0.249 | |
| Observe/evaluate the development of the child. | 39 (66.1) | 23 (82.1) | | 16 (61.5) | | 0.091 | |
| Identify problems in growth and development. | 45 (76.2) | 22 (91.5) | | 23 (85.2) | | 1.000 | |
| Talk about the importance of child development. | 53 (89.8) | | 27 (90.0) | 26 (92.9) | | 1.000 | |
| Explain mother/caregiver about developmental stages. | 39 (66.1) | | 21 (72.4) | | 18 (64.3) | | 0.509 |
| Stimulate the mother/caregiver to talk to the child. | 49 (83.0) | | 25 (86.2) | 24 (88.9) | | | 1.000 |
| Stimulate the mother/caregiver to read to the child. | 34 (57.6) | | 13 (46.4) | 21 (75.0) | | | **0.029** |
| Explain about the importance of reading for the child | 29 (49.1) | | 12 (41.4) | 17 (60.7) | | | 0.144 |
| Encourage mother/caregiver to tell stories to the child. | 36 (61.0) | | 14 (46.7) | 22 (78.6) | | | **0.012** |
| Encourage listening to music/sing with child. | 27 (45.7) | | 13 (43.3) | 14 (50.0) | | | 0.611 |
| Stimulate mother/caregiver to play with the child. | 49 (83.0) | | 23 (83.9) | 26 (92.9) | | | 0.428 |
| Encourage showing colored objects to the child. | 50 (84.7) | | 24 (77.4) | 26 (96.3) | | | 0.057 |
| Stimulate using moments of routine as stimuli moment. | 23 (38.9) | | 11 (37.9) | 12 (42.9) | | | 0.705 |

na not available. p-value could not be calculated due to 100 % correct answers.

| **Supplementary Table 4** Knowledge assessment of intervention group (n=31) pre-, post and one-year after training. | | | | | | |
| --- | --- | --- | --- | --- | --- | --- |
|  | Moments | | | |  |  |
| Correct answers | Pre-training | Post-training | One-year after training | | Pre- x Post-  p-value | Pre- x one-year  p-value |
|  | n (%) | n (%) | n (%) | |  |  |
| Risk factors for pregnant women |  |  | |  |  |  |
| Age under 19 years | 25 (80.6) | 25 (80.6) | | 21 (67.7) | 1.000 | 0.386 |
| Economic dependence | 1 (3.2) | 4 (12.9) | | 3 (9.7) | 0.354 | 0.523 |
| Marital problems | 3 (9.7) | 10 (32.3) | | 9 (29.0) | **0.029** | 0.077 |
| Low educational level | 3 (9.7) | 9 (29.0) | | 6 (19.4) | 0.054 | 0.156 |
| Twin pregnancy | 15 (48.4) | 20 (64.5) | | 18 (58.1) | 0.200 | 0.435 |
| Excessive weight gain | 26 (83.9) | 28 (90.3) | | 26 (83.9) | 0.707 | 0.806 |
| Blood pressure elevation | 28 (90.3) | 31 (100.0) | | 31 (100.0) | 0.238 | 0.104 |
| Warning signs in pregnant women |  |  | |  |  |  |
| Frequent nausea and vomiting | 8 (25.8) | 9 (29.0) | | 10 (32.3) | 0.776 | 0.855 |
| Vaginal discharge with bad smell | 26 (83.9) | 29 (93.5) | | 30 (96.8) | 0.425 | 0.265 |
| Fever | 25 (80.6) | 23 (74.2) | | 29 (93.5) | 0.544 | 0.121 |
| Bleeding | 27 (87.1) | 29 (93.5) | | 30 (96.8) | 0.671 | 0.494 |
| Edema on hands, feet and face | 21 (67.7) | 26 (83.9) | | 23 (74.2) | 0.138 | 0.334 |
| Absence of fetal movements | 25 (80.6) | 30 (96.8) | | 31 (100.0) | 0.104 | **0.015** |
| Risk factors/warning signs for child |  |  | |  |  |  |
| Birth weight below 2500g | 28 (90.3) | 31 (100.0) | | 31 (100.0) | 0.238 | 0.104 |
| Teen Mother | 24 (77.4) | 25 (80.6) | | 24 (77.4) | 0.755 | 0.938 |
| Late vaccination schedule | 28 (90.3) | 30 (96.8) | | 29 (93.5) | 0.301 | 0.868 |
| Death of a under 5 year sibling | 3 (9.7) | 11 (35.5) | | 7 (22.6) | **0.031** | 0.052 |
| Low parental education | 4 (12.9) | 9 (29.0) | | 5 (16.1) | 0.211 | 0.235 |
| Parents' financial problems | 1 (3.2) | 8 (25.8) | | 6 (19.4) | **0.026** | **0.045** |
| Delay in development | 22 (71.0) | 31 (100.0) | | 30 (96.8) | **0.002** | **<0.001** |
| Early weaning | 18 (58.1) | 26 (83.9) | | 27 (87.1) | **0.049** | **0.013** |
| Antenatal care |  |  | |  |  |  |
| Ideal period to start prenatal care | 28 (93.3) | 29 (93.5) | | 30 (96.8) | 1.000 | 0.868 |
| Number of prenatal consultations | 19 (61.3) | 30 (96.8) | | 24 (77.4) | **0.001** | **0.003** |
| Vaccination in pregnancy | 15 (50.0) | 25 (80.6) | | 24 (77.4) | **0.012** | **0.017** |
| Newborn care |  |  | |  |  |  |
| Moment of first home visit | 22 (71.0) | 29 (93.5) | | 27 (87.1) | **0.020** | **0.045** |
| Frequency of breastfeeding | 26 (83.9) | 31 (100.0) | | 30 (96.8) | 0.053 | **0.044** |
| Care for newborn with fever | 17 (54.8) | 17 (54.8) | | 18 (58.1) | 1.000 | 0.957 |
| Child care |  |  | |  |  |  |
| Nutrition in the first 2 years old | 25 (80.6) | 30 (96.8) | | 30 (96.8) | 0.104 | **0.046** |
| Vaccination in children | 29 (93.5) | 30 (96.8) | | 30 (96.8) | 1.000 | 1.000 |
| Prevention of accidents |  |  | |  |  |  |
| - Sleeping position | 20 (64.5) | 30 (96.8) | | 26 (83.9) | **0.003** | **0.004** |
| - Use of infant seats | 25 (80.6) | 26 (83.9) | | 27 (87.1) | 0.740 | 0.788 |
| - Risks of child walker | 19 (61.3) | 23 (74.2) | | 18 (58.1) | 0.277 | 0.373 |
| Child development |  |  | |  |  |  |
| Assessment of child development | 22 (71.0) | 23 (74.2) | | 24 (77.4) | 0.776 | 0.845 |
| Important stages for brain development | 24 (80.0) | 29 (93.5) | | 31 (100.0) | 0.147 | **0.011** |
| Practices that stimulate child development |  |  | |  |  |  |
| - Musical Experiences | 22 (73.3) | 30 (96.8) | | 30 (96.8) | **0.012** | **0.004** |
| - Massage | 17 (56.7) | 19 (61.3) | | 19 (61.3) | 0.714 | 0.914 |
| - Family Reading | 23 (76.7) | 31 (100.0) | | 29 (93.5) | **0.005** | **0.004** |
| - Playing | 24 (80.0) | 27 (87.1) | | 26 (83.9) | 0.508 | 0.754 |
| Risk factors for child development |  |  | |  |  |  |
| - Little social relationship | 24 (80.0) | 24 (77.4) | | 23 (74.2) | 0.806 | 0.864 |
| - Domestic Violence | 30 (100.0) | 31 (100.0) | | 29 (93.5) | na | 0.326 |
| - Parents' mental health problem | 19 (63.3) | 29 (93.5) | | 21 (67.7) | **0.004** | **0.013** |
| - Extended use of digital devices | 15 (50.0) | 26 (83.9) | | 24 (77.4) | **0.005** | **0.009** |
| - Parents' low level of education | 8 (26.7) | 16 (51.6) | | 9 (29.0) | **0.046** | 0.079 |
| Aims of home visits to mother and child health |  |  | |  |  |  |
| - Preparing for childbirth and postpartum | 23 (76.7) | 30 (96.8) | | 27 (87.1) | **0.026** | 0.059 |
| - Reduction of preterm and low birth weight | 18 (60.0) | 29 (93.5) | | 18 (60.0) | **0.002** | **0.003** |
| - Improvement of children's nutritional status | 28 (93.3) | 29 (93.5) | | 24 (77.4) | 1.000 | 0.101 |
| - Greater cognitive and emotional development | 16 (53.3) | 30 (96.8) | | 25 (80.6) | **<0.001** | **<0.001** |
| - Reduction of child violence | 20 (66.7) | 26 (83.9) | | 21 (67.7) | 0.119 | 0.236 |
| na not available. p-value could not be calculated due to 100 % correct answers. | | | | | | |

| **Supplementary Table 5** Knowledge assessment of intervention (n=31) and control (n=28) groups pre-and one-year after training. | | | | | | | |
| --- | --- | --- | --- | --- | --- | --- | --- |
| Correct answers | Pre-training scores | | |  | One-year after training scores | | |
|  | Intervention  n (%) | Control  n (%) | p-value |  | Intervention  n (%) | Control  n (%) | p-value |
| Risk factors for pregnant women |  |  |  |  |  |  |  |
| Age under 19 years | 25 (80.6) | 22 (78.6) | 0.843 |  | 21 (67.7) | 22 (78.6) | 0.350 |
| Economic dependence | 1 (3.2) | 1 (3.6) | 1.000 |  | 3 (9.7) | 2 (7.1) | 1.000 |
| Marital problems | 3 (9.7) | 4 (14.3) | 0.698 |  | 9 (29.0) | 1 (3.6) | **0.013** |
| Low educational level | 3 (9.7) | 5 (17.9) | 0.458 |  | 6 (19.4) | 4 (14.3) | 0.734 |
| Twin pregnancy | 15 (48.4) | 16 (57.1) | 0.501 |  | 18 (58.1) | 14 (50.0) | 0.535 |
| Excessive weight gain | 26 (83.9) | 22 (78.6) | 0.602 |  | 26 (83.9) | 25 (89.3) | 0.709 |
| Blood pressure elevation | 28 (90.3) | 27 (96.4) | 0.614 |  | 31 (100.0) | 28 (100.0) | na |
| Warning signs in pregnant women |  |  |  |  |  |  |  |
| Frequent nausea and vomiting | 8 (25.8) | 7 (25.0) | 0.943 |  | 10 (32.3) | 5 (17.9) | 0.205 |
| Vaginal discharge with bad smell | 26 (83.9) | 25 (89.3) | 0.709 |  | 30 (96.8) | 25 (89.3) | 0.337 |
| Fever | 25 (80.6) | 23 (82.1) | 0.883 |  | 29 (93.5) | 24 (85.7) | 0.409 |
| Bleeding | 27 (87.1) | 28 (100.0) | 0.114 |  | 30 (96.8) | 28 (100.0) | 1.000 |
| Edema on hands, feet and face | 21 (67.7) | 21 (75.0) | 0.539 |  | 23 (74.2) | 18 (64.3) | 0.409 |
| Absence of fetal movements | 25 (80.6) | 25 (89.3) | 0.477 |  | 31 (100.0) | 25 (89.3) | 0.101 |
| Risk factors/warning signs for child |  |  |  |  |  |  |  |
| Birth weight below 2500g | 28 (90.3) | 28 (100.0) | 0.239 |  | 31 (100.0) | 28 (100.0) | na |
| Teen Mother | 24 (77.4) | 16 (57.1) | 0.096 |  | 24 (77.4) | 14 (50.0) | **0.028** |
| Late vaccination schedule | 28 (90.3) | 21 (75.0) | 0.168 |  | 29 (93.5) | 21 (75.0) | 0.071 |
| Death of a under 5 year sibling | 3 (9.7) | 5 (17.9) | 0.458 |  | 7 (22.6) | 1 (3.6) | 0.055 |
| Low parental education | 4 (12.9) | 4 (14.3) | 1.000 |  | 5 (16.1) | 3 (10.7) | 0.709 |
| Parents' financial problems | 1 (3.2) | 2 (7.1) | 0.599 |  | 6 (19.4) | 1 (3.6) | 0.106 |
| Delay in development | 22 (71.0) | 15 (53.6) | 0.168 |  | 30 (96.8) | 24 (85.7) | 0.180 |
| Early weaning | 18 (58.1) | 19 (67.9) | 0.437 |  | 27 (87.1) | 16 (57.1) | **0.010** |
| Antenatal care |  |  |  |  |  |  |  |
| Ideal period to start prenatal care | 28 (93.3) | 25 (92.6) | 1.000 |  | 30 (96.8) | 27 (96.4) | 1.000 |
| Number of prenatal consultations | 19 (61.3) | 19 (67.9) | 0.888 |  | 24 (77.4) | 17 (60.7) | 0.164 |
| Vaccination in pregnancy | 15 (50.0) | 12 (46.2) | 0.142 |  | 24 (77.4) | 14 (50.0) | **0.028** |
| Newborn care |  |  |  |  |  |  |  |
| Moment of first home visit | 22 (71.0) | 23 (82.1) | 0.805 |  | 27 (87.1) | 26 (92.9) | 0.673 |
| Frequency of breastfeeding | 26 (83.9) | 20 (71.4) | 0.197 |  | 30 (96.8) | 23 (82.1) | 0.092 |
| Care for newborn with fever | 17 (54.8) | 21 (75.0) | 0.493 |  | 18 (58.1) | 18 (64.3) | 0.625 |
| Child care |  |  |  |  |  |  |  |
| Nutrition in the first 2 years old | 25 (80.6) | 20 (71.4) | 0.467 |  | 30 (96.8) | 24 (85.7) | 0.180 |
| Vaccination in children | 29 (93.5) | 27 (96.4) | 1.000 |  | 30 (96.8) | 27 (96.4) | 1.000 |
| Prevention of accidents |  |  |  |  |  |  |  |
| - Sleeping position | 20 (64.5) | 13 (46.4) | 0.163 |  | 26 (83.9) | 17 (60.7) | **0,046** |
| - Use of infant seats | 25 (80.6) | 17 (60.7) | 0.077 |  | 27 (87.1) | 12 (42.9) | **<0.001** |
| - Risks of child walker | 19 (61.3) | 17 (63.0) | 0.216 |  | 18 (58.1) | 15 (53.6) | 0.728 |
| Child development |  |  |  |  |  |  |  |
| Assessment of child development | 22 (71.0) | 20 (71.4) | 0.277 |  | 24 (77.4) | 21 (75.0) | 0.827 |
| Important stages for brain development | 24 (80.0) | 16 (59.3) | 0.156 |  | 31 (100.0) | 18 (64.3) | **<0.001** |
| Practices that stimulate child development |  |  |  |  |  |  |  |
| - Musical Experiences | 22 (73.3) | 21 (75.0) | 0.885 |  | 30 (96.8) | 22 (78.6) | **0.045** |
| - Massage | 17 (56.7) | 15 (53.6) | 0.813 |  | 19 (61.3) | 19 (67.9) | 0.786 |
| - Family Reading | 23 (76.7) | 19 (67.9) | 0.453 |  | 29 (93.5) | 20 (71.4) | **0.024** |
| - Playing | 24 (80.0) | 20 (71.4) | 0.446 |  | 26 (83.9) | 19 (67.9) | 0.149 |
| Risk factors for child development |  |  |  |  |  |  |  |
| - Little social relationship | 24 (80.0) | 21 (75.0) | 0.648 |  | 23 (74.2) | 19 (67.9) | 0.592 |
| - Domestic Violence | 30 (100.0) | 24 (85.7) | **0.048** |  | 29 (93.5) | 25 (89.3) | 0.661 |
| - Parents' mental health problem | 19 (63.3) | 12 (42.9) | 0.118 |  | 21 (67.7) | 11 (39.3) | **0.028** |
| - Extended use of digital devices | 15 (50.0) | 12 (42.9) | 0.586 |  | 24 (77.4) | 19 (67.9) | 0.409 |
| - Parents' low level of education | 8 (26.7) | 6 (21.4) | 0.641 |  | 9 (29.0) | 4 (14.3) | 0.172 |
| Aims of home visits to mother and child health |  |  |  |  |  |  |  |
| - Preparing for childbirth and postpartum | 23 (76.7) | 22 (78.6) | 0.862 |  | 27 (87.1) | 23 (82.1) | 0.723 |
| - Reduction of preterm and low birth weight | 18 (60.0) | 9 (32.1) | **0.034** |  | 18 (60.0) | 11 (39.3) | 0.150 |
| - Improvement of children's nutritional status | 28 (93.3) | 22 (78.6) | 0.138 |  | 24 (77.4) | 17 (60.7) | 0.164 |
| - Greater cognitive and emotional development | 16 (53.3) | 13 (46.4) | 0.599 |  | 25 (80.6) | 8 (28.6) | **<0.001** |
| - Reduction of child violence | 20 (66.7) | 13 (46.4) | 0.120 |  | 21 (67.7) | 8 (28.6) | **0.003** |
|  |  |  |  |  |  |  |  |

| **Supplementary Table 6** Attitudes assessment of intervention group (n=31) pre- and one-year after training. | | | | |
| --- | --- | --- | --- | --- |
|  | Moments | | |  |
| Behaviors | Pre-training | One year after training | | p-value |
|  | n (%) | | n (%) |  |
| Is punctual and assiduous in work activities. | 29 (96.7) | | 31 (100.0) | 0.492 |
| Perform work activities properly. | 28 (96.6) | | 31 (100.0) | 0.483 |
| Communicate appropriately with users. | 31 (100.0) | | 31 (100.0) | na |
| Use clear and simple language to communicate with users. | 31 (100.0) | | 31 (100.0) | na |
| Listen patiently to the situations brought by families. | 31 (100.0) | | 31 (100.0) | na |
| Respect the opinions, beliefs and religion of the users. | 31 (100.0) | | 31 (100.0) | na |
| Have good relationship with users. | 31 (100.0) | | 30 (96.8) | 1.000 |
| Have concerns for the health of users. | 31 (100.0) | | 31 (100.0) | na |
| Seek to update the knowledge about pregnant health. | 30 (96.8) | | 31 (100.0) | 1.000 |
| Is capable to provide assistance to pregnant women at home | 27 (87.1) | | 31 (100.0) | 0.113 |
| Seek to update the knowledge about child health. | 31 (100.0) | | 31 (100.0) | na |
| Is capable to provide assistance to children at home. | 29 (93.5) | | 29 (96.7) | 1.000 |
| Consider the importance of the cards of pregnant and child. | 31 (100.0) | | 31 (100.0) | na |
| When in doubt, seek support from other team members. | 31 (100.0) | | 31 (100.0) | na |
| Has good relationship with the other team members. | 31 (100.0) | | 31 (100.0) | na |
| Analyze and receive critics or suggestions. | 28 (96.6) | | 30 (100.0) | 0.492 |
| Reflects on your work process. | 29 (96.7) | | 31 (100.0) | 0.492 |
| Know the health indicators of the population that assist. | 28 (96.6) | | 29 (93.5) | 1.000 |
| Feel happy and satisfied her/his your work. | 28 (90.3) | | 31 (100.0) | 0.238 |
| Perceive satisfactory results of your work. | 29 (93.5) | | 30 (96.8) | 1.000 |

na not available. p-value could not be calculated due to 100 % correct answers.

| **Supplementary Table 7** Attitudes assessment of intervention (n=31) and control (n=28) groups pre-and one-year after training. | | | | | | | | |
| --- | --- | --- | --- | --- | --- | --- | --- | --- |
| Behaviors | Pre-training scores | | | |  | One-year after training scores | | |
|  | Intervention  n (%) | Control  n (%) | p-value | |  | Intervention  n (%) | Control  n (%) | p-value |
| Is punctual and assiduous in work activities. | 29 (96.7) | 24 (88.9) | | 0.336 |  | 31 (100.0) | 22 (81.5) | **0.018** |
| Perform work activities properly. | 28 (96.6) | 25 (92.6) | | 0.605 |  | 31 (100.0) | 26 (96.3) | 0.466 |
| Communicate appropriately with users. | 31 (100.0) | 26 (96.3) | | 0.466 |  | 31 (100.0) | 26 (96.3) | 0.466 |
| Use clear and simple language to communicate with users. | 31 (100.0) | 28 (100.0) | | na |  | 31 (100.0) | 28 (100.0) | na |
| Listen patiently to the situations brought by families. | 31 (100.0) | 26 (96.3) | | 0.466 |  | 31 (100.0) | 28 (100.0) | na |
| Respect the opinions. beliefs. and religion of the users. | 31 (100.0) | 26 (96.3) | | 0.466 |  | 31 (100.0) | 28 (100.0) | na |
| Have good relationship with users. | 31 (100.0) | 25 (92.6) | | 0.220 |  | 30 (96.8) | 26 (96.3) | 1.000 |
| Have concerns for the health of users. | 31 (100.0) | 26 (96.3) | | 0.466 |  | 31 (100.0) | 26 (96.3) | 0.466 |
| Seek to update the knowledge about pregnant health. | 30 (96.8) | 23 (85.2) | | 0.173 |  | 31 (100.0) | 24 (88.9) | 0.095 |
| Is capable to provide assistance to pregnant women at home | 27 (87.1) | 23 (85.2) | | 1.000 |  | 31 (100.0) | 22 (81.5) | 0.019 |
| Seek to update the knowledge about child health. | 31 (100.0) | 28 (100.0) | | na |  | 31 (100.0) | 24 (88.9) | 0.095 |
| Is capable to provide assistance to children at home. | 29 (93.5) | 22 (85.1) | | 0.233 |  | 29 (96.7) | 24 (88.9) | 0.336 |
| Consider the importance of the cards of pregnant and child. | 31 (100.0) | 28 (100.0) | | na |  | 31 (100.0) | 26 (96.3) | 0.466 |
| When in doubt. seek support from other team members. | 31 (100.0) | 28 (100.0) | | na |  | 31 (100.0) | 28 (100.0) | na |
| Has good relationship with the other team members. | 31 (100.0) | 26 (96.3) | | 0.466 |  | 31 (100.0) | 26 (96.3) | 0.466 |
| Analyze and receive critics or suggestions. | 28 (96.6) | 22 (85.1) | | 0.096 |  | 30 (100.0) | 28 (100.0) | na |
| Reflects on your work process. | 29 (96.7) | 25 (92.6) | | 0.599 |  | 31 (100.0) | 28 (100.0) | na |
| Know the health indicators of the population that assist. | 28 (96.6) | 20 (74.1) | | **0.023** |  | 29 (93.5) | 25 (92.6) | 1.000 |
| Feel happy and satisfied her/his your work. | 28 (90.3) | 19 (70.4) | | 0.053 |  | 31 (100.0) | 24 (88.9) | 0.095 |
| Perceive satisfactory results of your work. | 29 (93.5) | 26 (96.3) | | 1.000 |  | 30 (96.8) | 25 (92.6) | 0,593 |

na not available. p-value could not be calculated due to 100 % correct answers.

| **Supplementary Table 8** Practices assessment of intervention group (n=31) pre- and one-year after training. | | | | |
| --- | --- | --- | --- | --- |
|  | Moments | | |  |
| Tasks | Pre-training | One year after training | | p-value |
|  | n (%) | | n (%) |  |
| Task performed at home visits to pregnant women |  | |  |  |
| Identify pregnant women at risk. | 23 (76.7) | | 28 (90.3) | 0.182 |
| Identify warning signs in pregnancy. | 27 (90.0) | | 31 (100.0) | 0.113 |
| Explain about warning signs to get health care. | 29 (96.7) | | 31 (100.0) | 0.492 |
| Forward pregnant women with problems for the unit. | 31 (100.0) | | 29 (93.5) | 0.492 |
| Talk about the importance of prenatal care. | 31 (100.0) | | 31 (100.0) | na |
| Motivate pregnant women to attend consultations. | 31 (100.0) | | 31 (100.0) | na |
| Encourage the father's participation in prenatal care. | 16 (53.3) | | 27 (87.1) | **0.004** |
| Monitor the prenatal follow-up. | 30 (96.8) | | 31(100.0) | 1.000 |
| Verify the fill of the pregnant woman's card. | 23 (76.7) | | 27 (87.1) | 0.289 |
| Encourage the reading of the pregnant woman's card. | 12 (41.4) | | 25 (80.6) | **0.002** |
| Talk about the importance of prenatal exams. | 28 (90.3) | | 31 (100.0) | 0.238 |
| Inform about vaccination schedule in pregnancy | 28 (90.3) | | 29 (93.5) | 1.000 |
| Inform about correct nutrition in pregnancy | 25 (80.6) | | 25 (80.6) | 1.000 |
| Inform about iron replacement | 20 (66.7) | | 26 (83.9) | 0.119 |
| Orient about physical activity in pregnancy. | 11 (36.7) | | 18 (62.1) | 0.051 |
| Alert on alcohol and drug risks in pregnancy. | 22 (71.0) | | 30 (96.8) | **0.006** |
| Explain about child development in pregnancy | 23 (79.3) | | 30 (96.8) | **0.049** |
| Stimulate mother to sing for the baby in the belly. | 11 (37.9) | | 24 (77.4) | **0.002** |
| Stimulate the mother to talk to the baby in the belly. | 21 (67.7) | | 27 (90.0) | **0.034** |
| Talk about the benefits of normal birth. | 25 (86.0) | | 24 (77.4) | 0.755 |
| Explain about the signs of labor. | 23 (74.2) | | 25 (80.6) | 0.544 |
| Explain about labor and childbirth. | 16 (53.3) | | 17 (54.8) | 0.906 |
| Provide explanations about postpartum. | 19 (63.3) | | 24 (77.4) | 0.228 |
| Talk about the benefits of breastfeeding. | 29 (93.5) | | 29 (93.5) | 1.000 |
| Orient about future contraception and family planning. | 22 (73.3) | | 30 (96.8) | **0.012** |
| Tasks performed at home visits to mother and child. |  | |  |  |
| Identify children at risk. | 21 (72.4) | | 30 (96.8) | **0.011** |
| Identify signs of child suffering violence. | 21 (72.4) | | 29 (93.5) | **0.039** |
| Observe relationship of child and parents/caregivers. | 23 (79.3) | | 29 (93.5) | 0.140 |
| Encourage the father's involvement in childcare. | 22 (75.9) | | 25 (80.6) | 0.653 |
| Ask mother/caregiver about problem with the child. | 31 (100.0) | | 30 (96.8) | 1.000 |
| Explain about warning signs to get child to health care. | 29 (100.0) | | 30 (96.8) | 1.000 |
| Verify the fill of the child's card. | 31 (100.0) | | 30 (96.8) | 1.000 |
| Instruct mother/caregiver about the child's card. | 27 (93.1) | | 31 (100.0) | 0.229 |
| Inform about child vaccination schedule. | 30 (100.0) | | 31 (100.0) | na |
| Talk about the benefits of breastfeeding. | 28 (96.6) | | 30 (96.8) | 1.000 |
| Orient about breastfeeding position and latch-on. | 28 (93.3) | | 31 (100.0) | 0.238 |
| Observe attachment and correct errors. | 27 (90.0) | | 28 (90.3) | 1.000 |
| Identify problems in breastfeeding. | 25 (86.2) | | 31 (100.0) | **0.049** |
| Talk about breast care during breastfeeding. | 29 (96.7) | | 31 (100.0) | 0.492 |
| Orient about weaning and storage of breast milk. | 19 (63.6) | | 24 (77.4) | 0.228 |
| Alert on risks of baby bottles and pacifiers. | 28 (93.3) | | 31 (100.0) | 0.238 |
| Inform about correct child nutrition. | 24 (82.8) | | 29 (93.5) | 0.247 |
| Inform about quantity and quality of child food. | 26 (89.7) | | 29 (93.5) | 0.666 |
| Orient about child meal times. | 23 (85.2) | | 31 (100.0) | **0.041** |
| Explain about correct child hygiene habits. | 28 (96.6) | | 31 (100.0) | 0.483 |
| Explain about oral hygiene of the child. | 27 (93.1) | | 31 (100.0) | 0.229 |
| Inform about accident prevention. | 26 (89.7) | | 30 (96.8) | 0.346 |
| Ask mother/caregiver about the child's development. | 24 (85.7) | | 29 (93.5) | 0.409 |
| Observe/evaluate the development of the child. | 23 (82.1) | | 28 (90.3) | 0.458 |
| Identify problems in growth and development. | 22 (91.5) | | 31 (100.0) | **0.018** |
| Talk about the importance of child development. | 27 (90.0) | | 29 (93.5) | 0.671 |
| Explain mother/caregiver about developmental stages. | 21 (72.4) | | 25 (80.6) | 0.451 |
| Stimulate the mother/caregiver to talk to the child. | 25 (86.2) | | 29 (93.5) | 0.417 |
| Stimulate the mother/caregiver to read to the child. | 13 (46.4) | | 20 (66.7) | 0.120 |
| Explain about the importance of reading for the child | 12 (41.4) | | 19 (61.3) | 0.123 |
| Encourage mother/caregiver to tell stories to the child. | 14 (46.7) | | 22 (71.0) | 0.054 |
| Encourage listening to music/sing with child. | 13 (43.3) | | 22 (71.0) | **0.029** |
| Stimulate mother/caregiver to play with the child. | 23 (83.9) | | 28 (90.3) | 0.707 |
| Encourage showing colored objects to the child. | 24 (77.4) | | 23 (74.2) | 1.000 |
| Stimulate using moments of routine as stimuli moment. | 11 (37.9) | | 20 (64.5) | **0.039** |

na not available. p-value could not be calculated due to 100 % correct answers.

| **Supplementary Table 9** Practices assessment of intervention (n=31) and control (n=28) groups pre-and one-year after training. | | | | | | | | |
| --- | --- | --- | --- | --- | --- | --- | --- | --- |
| Tasks | Pre-training scores | | | |  | One-year after training scores | | |
|  | Intervention  n (%) | Control  n (%) | p-value | |  | Intervention  n (%) | Control  n (%) | p-value |
| Task performed at home visits to pregnant women |  |  | |  |  |  |  |  |
| Identify pregnant women at risk. | 23 (76.7) | 22 (81.5) | | 0.656 |  | 28 (90.3) | 20 (71.4) | 0.063 |
| Identify warning signs in pregnancy. | 27 (90.0) | 24 (85.7) | | 0.701 |  | 31 (100.0) | 24 (85.7) | **0.045** |
| Explain about warning signs to get health care. | 29 (96.7) | 25 (89.3) | | 0.344 |  | 31 (100.0) | 26 (92.9) | 0.221 |
| Forward pregnant women with problems for the unit. | 31 (100.0) | 28 (100.0) | | na |  | 29 (93.5) | 28 (100.0) | 0.493 |
| Talk about the importance of prenatal care. | 31 (100.0) | 26 (92.9) | | 0.221 |  | 31 (100.0) | 28 (100.0) | na |
| Motivate pregnant women to attend consultations. | 31 (100.0) | 28 (100.0) | | na |  | 31 (100.0) | 28 (100.0) | na |
| Encourage the father's participation in prenatal care. | 16 (53.3) | 15 (53.6) | | 0.986 |  | 27 (87.1) | 20 (71.4) | 0.135 |
| Monitor the prenatal follow-up. | 30 (96.8) | 28 (100.0) | | 1.000 |  | 31(100.0) | 24 (88.9) | 0.095 |
| Verify the fill of the pregnant woman's card. | 23 (76.7) | 21 (75.0) | | 0.882 |  | 27 (87.1) | 25 (89.3) | 1.000 |
| Encourage the reading of the pregnant woman's card. | 12 (41.4) | 13 (46.4) | | 0.701 |  | 25 (80.6) | 21 (75.0) | 0.601 |
| Talk about the importance of prenatal exams. | 28 (90.3) | 27 (96.4) | | 0.614 |  | 31 (100.0) | 28 (100.0) | na |
| Inform about vaccination schedule in pregnancy | 28 (90.3) | 27 (96.4) | | 0.614 |  | 29 (93.5) | 24 (88.9) | 0.656 |
| Inform about correct nutrition in pregnancy | 25 (80.6) | 19 (73.1) | | 0.498 |  | 25 (80.6) | 22 (78.6) | 0.843 |
| Inform about iron replacement | 20 (66.7) | 18 (66.7) | | 1.000 |  | 26 (83.9) | 24 (88.9) | 0.712 |
| Orient about physical activity in pregnancy. | 11 (36.7) | 10 (37.0) | | 0.977 |  | 18 (62.1) | 13 (46.4) | 0.236 |
| Alert on alcohol and drug risks in pregnancy. | 22 (71.0) | 21 (75.0) | | 0.728 |  | 30 (96.8) | 24 (88.9) | 0.180 |
| Explain about child development in pregnancy | 23 (79.3) | 26 (92.9) | | 0.253 |  | 30 (96.8) | 26 (92.9) | 0.599 |
| Stimulate mother to sing for the baby in the belly. | 11 (37.9) | 9 (36.0) | | 0.884 |  | 24 (77.4) | 15 (53.6) | 0.097 |
| Stimulate the mother to talk to the baby in the belly. | 21 (67.7) | 16 (57.1) | | 0.401 |  | 27 (90.0) | 18 (64.3) | **0.019** |
| Talk about the benefits of normal birth. | 25 (86.0) | 24 (85.7) | | 0.734 |  | 24 (77.4) | 22 (78.6) | 0.915 |
| Explain about the signs of labor. | 23 (74.2) | 23 (82.1) | | 0.462 |  | 25 (80.6) | 20 (71.4) | 0.406 |
| Explain about labor and childbirth. | 16 (53.3) | 15 (53.6) | | 0.986 |  | 17 (54.8) | 11 (39.3) | 0.232 |
| Provide explanations about postpartum. | 19 (63.3) | 20 (71.4) | | 0.512 |  | 24 (77.4) | 19 (67.9) | 0.409 |
| Talk about the benefits of breastfeeding. | 29 (93.5) | 28 (100.0) | | 0.493 |  | 29 (93.5) | 28 (100.0) | 0.493 |
| Orient about future contraception and family planning. | 22 (73.3) | 26 (92.9) | | 0.081 |  | 30 (96.8) | 27 (96.4) | 1.000 |
| Tasks performed at home visits to mother and child. |  |  | |  |  |  |  |  |
| Identify children at risk. | 21 (72.4) | 21 (75.0) | | 0.825 |  | 30 (96.8) | 23 (82.1) | 0.092 |
| Identify signs of child suffering violence. | 21 (72.4) | 20 (71.4) | | 0.934 |  | 29 (93.5) | 20 (71.4) | **0.036** |
| Observe relationship of child and parents/caregivers. | 23 (79.3) | 24 (85.7) | | 0.730 |  | 29 (93.5) | 22 (78.6) | 0.134 |
| Encourage the father's involvement in childcare. | 22 (75.9) | 21 (75.0) | | 0.940 |  | 25 (80.6) | 17 (60.7) | 0.091 |
| Ask mother/caregiver about problem with the child. | 31 (100.0) | 28 (100.0) | | na |  | 30 (96.8) | 27 (96.4) | 1.000 |
| Explain about warning signs to get child to health care. | 29 (100.0) | 25 (96.2) | | 0.473 |  | 30 (96.8) | 26 (92.9) | 0.599 |
| Verify the fill of the child's card. | 31 (100.0) | 26 (92.9) | | 0.221 |  | 30 (96.8) | 28 (100.0) | 1.000 |
| Instruct mother/caregiver about the child's card. | 27 (93.1) | 24 (88.9) | | 0.664 |  | 31 (100.0) | 25 (89.3) | 0.101 |
| Inform about child vaccination schedule. | 30 (100.0) | 26 (96.3) | | 0.474 |  | 31 (100.0) | 27 (96.4) | 0.475 |
| Talk about the benefits of breastfeeding. | 28 (96.6) | 28 (100.0) | | 1.000 |  | 30 (96.8) | 26 (92.9) | 0.599 |
| Orient about breastfeeding position and latch-on. | 28 (93.3) | 27 (96.4) | | 1.000 |  | 31 (100.0) | 25 (89.3) | 0.101 |
| Observe attachment and correct errors. | 27 (90.0) | 25 (92.6) | | 1.000 |  | 28 (90.3) | 23 (82.1) | 0.458 |
| Identify problems in breastfeeding. | 25 (86.2) | 24 (85.7) | | 1.000 |  | 31 (100.0) | 25 (89.3) | 0.101 |
| Talk about breast care during breastfeeding. | 29 (96.7) | 25 (92.6) | | 0.599 |  | 31 (100.0) | 26 (92.9) | 0.221 |
| Orient about weaning and storage of breast milk. | 19 (63.6) | 21 (75.0) | | 0.337 |  | 24 (77.4) | 22 (78.6) | 0.915 |
| Alert on risks of baby bottles and pacifiers. | 28 (93.3) | 28 (100.0) | | 0.492 |  | 31 (100.0) | 27 (96.4) | 0.475 |
| Inform about correct child nutrition. | 24 (82.8) | 23 (88.5) | | 0.708 |  | 29 (93.5) | 27 (96.4) | 1.000 |
| Inform about quantity and quality of child food. | 26 (89.7) | 25 (92.6) | | 1.000 |  | 29 (93.5) | 25 (89.3) | 0.661 |
| Orient about child meal times. | 23 (85.2) | 23 (85.2) | | 1.000 |  | 31 (100.0) | 23 (82.1) | **0.020** |
| Explain about correct child hygiene habits. | 28 (96.6) | 27 (100.0) | | 1.000 |  | 31 (100.0) | 26 (92.9) | 0.221 |
| Explain about oral hygiene of the child. | 27 (93.1) | 25 (92.6) | | 1.000 |  | 31 (100.0) | 26 (92.9) | 0.221 |
| Inform about accident prevention. | 26 (89.7) | 24 (88.9) | | 1.000 |  | 30 (96.8) | 21 (75.0) | **0.022** |
| Ask mother/caregiver about the child's development. | 24 (85.7) | 19 (73.1) | | 0.249 |  | 29 (93.5) | 23 (82.1) | 0.240 |
| Observe/evaluate the development of the child. | 23 (82.1) | 16 (61.5) | | 0.091 |  | 28 (90.3) | 23 (82.1) | 0.458 |
| Identify problems in growth and development. | 22 (91.5) | 23 (85.2) | | 1.000 |  | 31 (100.0) | 20 (71.4) | **0.001** |
| Talk about the importance of child development. | 27 (90.0) | 26 (92.9) | | 1.000 |  | 29 (93.5) | 25 (89.3) | 0.661 |
| Explain mother/caregiver about developmental stages. | 21 (72.4) | 18 (64.3) | | 0.509 |  | 25 (80.6) | 17 (60.7) | 0.091 |
| Stimulate the mother/caregiver to talk to the child. | 25 (86.2) | 24 (88.9) | | 1.000 |  | 29 (93.5) | 26 (92.9) | 1.000 |
| Stimulate the mother/caregiver to read to the child. | 13 (46.4) | 21 (75.0) | | **0.029** |  | 20 (66.7) | 21 (75.0) | 0.486 |
| Explain about the importance of reading for the child | 12 (41.4) | 17 (60.7) | | 0.144 |  | 19 (61.3) | 19 (67.9) | 0.599 |
| Encourage mother/caregiver to tell stories to the child. | 14 (46.7) | 22 (78.6) | | **0.012** |  | 22 (71.0) | 21 (75.0) | 0.728 |
| Encourage listening to music/sing with child. | 13 (43.3) | 14 (50.0) | | 0.611 |  | 22 (71.0) | 18 (64.3) | 0.583 |
| Stimulate mother/caregiver to play with the child. | 23 (83.9) | 26 (92.9) | | 0.428 |  | 28 (90.3) | 26 (92.9) | 1.000 |
| Encourage showing colored objects to the child. | 24 (77.4) | 26 (96.3) | | 0.057 |  | 23 (74.2) | 20 (71.4) | 0.811 |
| Stimulate using moments of routine as stimuli moment. | 11 (37.9) | 12 (42.9) | | 0.705 |  | 20 (64.5) | 15 (53.6) | 0.393 |

na not available. p-value could not be calculated due to 100 % correct answers.
